# Associations of diet with infectious diseases in UK Biobank

**DOI:** 10.7555/JBR.37.20230319

**Published:** 2024-05-29

**Authors:** Junlan Tu, Xuehong Cai, Yifan Wang, Xiangyu Ye, Meijie Yu, Sheng Yang, Rongbin Yu, Peng Huang

**Affiliations:** 1 Department of Epidemiology, Center for Global Health, School of Public Health, National Vaccine Innovation Platform, Nanjing Medical University, Nanjing, Jiangsu 211166, China; 2 Department of Infectious Disease, Jurong Hospital Affiliated to Jiangsu University, Jurong, Jiangsu 212400, China; 3 Department of Biostatistics, Center for Global Health, School of Public Health, National Vaccine Innovation Platform, Nanjing Medical University, Nanjing, Jiangsu 211166, China

**Keywords:** infectious diseases, diet, food groups, UK Biobank, mediation analysis

## Abstract

The current study used multivariable logistic regression analysis to investigate associations between the intake frequencies of 13 food groups (or four diet groups) and infectious diseases. The analysis included 487849 participants from the UK Biobank, with 75209 participants diagnosed with infectious diseases. Participants reporting the highest intake frequency of processed meat (odds ratio [OR] = 1.0964, 95% confidence interval [CI]: 1.0622–1.1318) and red meat (OR = 1.0895, 95% CI: 1.0563–1.1239) had a higher risk of infectious diseases, compared with those with the lowest intake frequency. Consuming fish 2.0–2.9 times (OR = 0.8221, 95% CI: 0.7955–0.8496), cheese ≥ 5.0 times (OR = 0.882 2, 95% CI: 0.855 9–0.9092), fruit 3.0–3.9 servings (OR = 0.8867, 95% CI: 0.8661–0.9078), and vegetables 2.0–2.9 servings (OR = 0.9372, 95% CI: 0.9189–0.9559) per week were associated with a lower risk of infection. Low meat-eaters (OR = 0.9404, 95% CI: 0.9243–0.9567), fish-eaters (OR = 0.8391, 95% CI: 0.7887–0.8919), and vegetarians (OR = 0.9154, 95% CI: 0.8561–0.9778) had a lower risk of infectious diseases, compared with regular meat-eaters. The mediation analysis revealed that glycosylated hemoglobin, white blood cell count, and body mass index served as the mediators in the associations between diet and infectious diseases. The current study indicates that the intake frequency of food groups is a risk factor for infectious diseases, and fish-eaters have a lower risk of infection.

## Introduction

Infectious diseases are a global threat that contributes to excess morbidity and mortality annually, with a persistent potential for unpredictable pandemics, leading to a severe burden of diseases ^[ 1– 2] ^. Particularly, the emerging infectious coronavirus disease 2019 (COVID-19), which has been rampant over the past three years, served as a lesson to countries worldwide and forced us to refocus our attention on infectious diseases ^[ 3] ^. Therefore, it is of public health importance to investigate the pathways or factors that may prevent infectious diseases.

Infectious diseases are generally caused by microorganisms, yet exposure to the pathogens is a necessary but insufficient condition for infectious diseases ^[ 4] ^. The pandemic of infectious diseases is influenced by natural and social factors, with the social factors being notably more significant ^[ 5– 6] ^. Diet, as a crucial lifestyle factor, plays a role in the occurrence and epidemic of diseases, and a high number of disability-adjusted life years have been reported to be attributable to dietary risk factors ^[ 7] ^. To date, research on the associations between diet and diseases has predominantly focused on non-communicable diseases, such as cancers, cardiovascular diseases, metabolic diseases, and obesity ^[ 8– 11] ^. Numerous studies have demonstrated that diet is a crucial risk factor in non-communicable diseases. However, studies investigating the associations between diet and infectious diseases remain insufficient, and previous studies have only focused on a single infection ^[ 12– 13] ^. Therefore, it is necessary and essential to conduct a prospective study with a large sample size to investigate the association between diet and the incidence of various infectious diseases.

Studies have demonstrated a strong association between diet and body metabolism. The types and quantities of food consumed regularly may significantly affect the metabolism, such as blood lipids and glycemic indices, including triglycerides (TG) and glycosylated hemoglobin (HbA1c) ^[ 14– 15] ^. While studies have illuminated the critical role of body metabolism in various diseases, its involvement in the associations between diet and infectious diseases remains uninvestigated ^[ 16– 17] ^. Furthermore, a balanced diet profoundly influences nutritional status of the body, and maintaining optimal nutrition may prevent numerous diseases and promote overall health ^[ 18– 19] ^. Therefore, identifying indicators, such as body mass index (BMI), body fat percentage (BFP), and waist-to-hip ratio (WHR), that reflect the body's nutritional status, and investigating their roles in the associations between diet and infectious diseases may facilitate the prevention of infectious diseases. In addition, the immune level may also affect susceptibility to infectious diseases and their outcomes, so immune-related indicators, such as white blood cell count (WBC), should also be taken into consideration ^[ 20] ^.

The United Kingdom (UK) Biobank is a large-scale research resource that contains lifestyle and health information from over 500000 UK participants ^[ 21] ^. The UK Biobank recruitment center collected dietary information from participants, including the intake frequencies of main foods or food groups using the UK Biobank short food-frequency touchscreen questionnaire. Additionally, disease information was obtained through hospital diagnostic records. These initial conditions provide a unique opportunity to investigate hypotheses about diet and infectious diseases in the UK.

Here, we used data from the UK Biobank to evaluate the associations between infectious diseases (common subgroups: respiratory infectious diseases, digestive infectious diseases, and blood or sexually transmitted infectious diseases) and the consumption of various food groups. Subsequently, we conducted a series of subgroup analyses to assess these associations across sex and ethnic subpopulations. In addition, we assessed the associations of four common diets with infectious diseases and investigated the mediation effects of potential intermediate variables.

## Subjects and methods

### Study population

**Figure 1:**
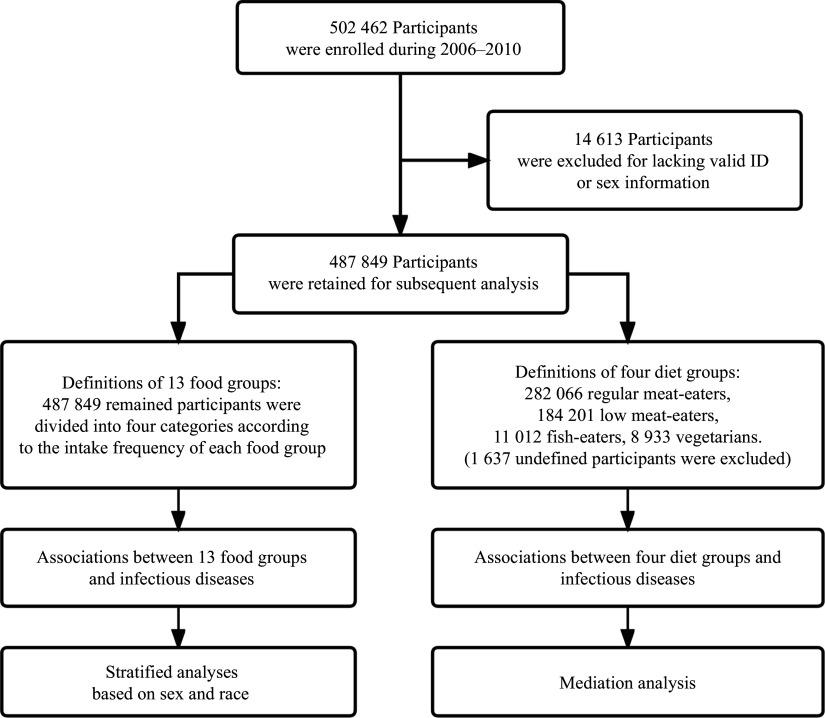
The flow diagram of participant selection in the UK Biobank.

The UK Biobank is a large-scale research resource that contains half a million UK participants aged 40–70 years, recruited from 22 assessment centers between 2006 and 2010 ^[ 22] ^. The UK Biobank has the ethical approval from the North West Multi-center Research Ethics Committee. Informed consent was obtained from each subject or their legal guardians. We used data collected up to March 26, 2021. There were 502462 participants in this time period, and we excluded the individuals with a mismatch between genetic and social sex, as well as those who were redacted, thus lacking a corresponding ID in the UK Biobank, following the established protocols ^[ 5, 23] ^. As a result, 487849 participants in the UK Biobank were retained for subsequent analyses ( ***Fig. 1***).

## Assessment of dietary intakes

The participants included in the UK Biobank were invited to complete a touchscreen questionnaire at the local assessment centers. This touchscreen questionnaire included 29 questions about diet, most of which were about the intake frequencies of main foods or food groups, including processed meat, poultry, beef, mutton, pork, oily fish, non-oily fish, cooked vegetables, raw vegetables, fresh fruit, dried fruit, cheese, bread, cereals, tea, water, and alcohol. This questionnaire required the participants to report their average intake of each type of food over the last year. In the current study, we combined two or three foods of the same kind into one food group and then grouped participants into four categories according to the distribution of data following the reported study ^[ 24] ^. The cut-offs for the categories were chosen to ensure a reasonable and similar number of the participants in each group. For example, by combining beef, mutton, and pork into the "Red meat" food group, we then divided the participants into four categories: "< 1 time per week", "1.0–1.9 times per week", "2.0–2.9 times per week", and "≥ 3.0 times per week". In addition, oily fish and non-oily fish were combined into the "Fish" group; cooked vegetables and raw vegetables were combined into the "Vegetables" group; fresh fruit and dried fruit were combined into the "Fruit" group; processed meat and red meat were combined into the "Red and processed meat" group. We divided main foods or food groups into animal (including processed meat, poultry, red meat, red and processed meat, fish, and cheese) and other food groups (fruits, vegetables, bread, cereals, tea, water, and alcohol). The calculation methods and more details of the food groups are shown in ***Supplementary Table 1*** (available online).

## Definition of outcome

In the current study, infectious diseases diagnosed after recruitment (the dietary survey) were defined according to diagnosis records in the UK Biobank, coded by the International Classification of Diseases, version 10 (ICD10) and version 9 (ICD9) ( ***Supplementary Table 2*** [available online]). Referring to the coding terms, we identified a total of 75209 (15.42%) cases of infectious disease. We further classified these into three subtypes of infectious diseases to investigate the association between dietary factors and common types of infectious diseases: (1) respiratory infectious diseases with 2663 (3.54%) cases; (2) digestive infectious diseases (excluding food poisoning) with 18678 (24.83%) cases; and (3) blood or sexually transmitted infectious diseases with 1051 (1.40%) cases.

## Definition of four diet groups

In the current study, we additionally categorized the participants into four common diet groups according to their reported consumption of meat and fish following the literature ^[ 25] ^: (1) regular meat-eaters (those who consumed meat including processed meat, red meat, and poultry more than five times a week); (2) low meat-eaters (those who consumed meat less than five times per week); (3) fish-eaters (those who consumed fish but never meat); and (4) vegetarians (those who never consumed any meat or fish). It should be noted that both regular meat-eaters and low meat-eaters may also have a habit of eating fish (consuming fish more than zero times a week).

## Statistical analysis

Continuous variables were presented as mean ± standard deviation or median (quartile) depending on the data distribution, and categorical variables were presented as counts (percentage). The baseline characteristics of the two groups were compared using the unpaired, 2-tailed Student's *t*-test or Wilcoxon test for continuous variables, and the *χ* ^2^ test was used for categorical variables.

Multivariable logistic regression was used to estimate associations between infectious diseases and reported intake of food or food groups. We treated sex, age, ethnicity, assessment center, activity, Townsend deprivation index (TDI), and education as covariates, and reported adjusted odds ratios (ORs) with 95% confidence intervals (CIs). Definitions of these covariates are listed in ***Supplementary Table 3*** (available online). Additionally, we treated the dietary exposures as the continuous trend variables in the multivariable logistic models to assess the linear trend.

We conducted a series of sensitivity analyses for infectious diseases. First, we excluded 55318 participants who reported that they had made a major change to their diet in the past five years because of illness or preferred not to answer the question ("Have you made any major changes to your diet in the last five years?"), and re-analyzed the associations between infectious disease incidence and the consumption of food groups in the remaining 432531participants (adjusting for sex, age, ethnicity, assessment center, activity, TDI, and education). Second, we performed a stratified analysis by sex. To test the heterogeneity of the main associations by sex, we compared models with and without an interaction term for the main exposure (as a continuous trend variable) and sex, and evaluated the significance using the likelihood ratio test. Third, given that ethnicity may affect the associations, we assessed the associations between infectious diseases and the intake of food groups in four ethnic subgroups ( *i.e.*, White, Asian or Asian British, Black or Black British, and Mixed or others).

We also performed a mediation analysis to estimate the proportion mediated by metabolic-related biomarkers ( *e.g.*, TG, blood glucose, and HbA1c), immune-related indicator (WBC), nutritional status-related indicators (BMI, BFP, and WHR), and body fat distribution-related indicators (whole body fat mass, arm fat mass, leg fat mass, and trunk fat mass) for the associations between diet groups and infectious diseases. In conducting the mediation analysis, both linear regression and logistic regression were used, and all regression analyses were adjusted for sex, age, ethnicity, assessment center, activity, TDI, and education.

All analyses were performed using R (version 4.1.2, R Foundation for Statistical Computing). *P* < 0.05 was considered statistically significant.

## Results

### Population characteristics

The current study included 487849 participants from the UK Biobank. ***Table 1*** shows the baseline population characteristics. The mean age of these participants was 56.54 (± 8.09) years, with 223312 (45.77%) men and 264537 (54.23%) women.

**Table 1:** Population characteristics of participants from the UK Biobank

Variables	Description		All ( *N*=487849)	*P*	
	Non-infection ( *n*=412640)	Infection ( *n*=75209)			
Age [years, *n* (%)]				<0.000 1	
<45	45274 (10.97)	4899 (6.51)	50173 (10.28)		
45–49	57503 (13.94)	6528 (8.68)	64031 (13.13)		
50–54	65006 (15.75)	9006 (11.97)	74012 (15.17)		
55–59	75759 (18.36)	12346 (16.42)	88105 (18.06)		
60–64	97720 (23.68)	20473 (27.22)	118193 (24.23)		
≥65	71378 (17.30)	21957 (29.20)	93335 (19.13)		
Male [ *n* (%)]	186190 (45.12)	37122 (49.36)	223312 (45.77)	<0.000 1	
White ethnicity or race [ *n* (%)]	389308 (94.35)	70433 (93.65)	459741 (94.24)	<0.0001	
TDI [median (quartile)]	−2.22 (−3.67, 0.35)	−1.66 (−3.39, 1.50)	−2.15 (−3.65, 0.53)	<0.000 1	
BMI [kg/m ^2^, *n* (%)]				<0.0001	
<18.5	9637 (2.34)	1731 (2.32)	11368 (2.34)		
18.5≤BMI<25	130642 (31.76)	18731 (25.14)	149373 (30.74)		
25≤BMI<30	176513 (42.91)	30241 (40.59)	206754 (42.55)		
≥30	94571 (22.99)	23808 (31.95)	118379 (24.37)		
Income [£, *n* (%)]				<0.0001	
<18000	20674 (5.83)	1848 (2.99)	22522 (5.41)		
18000–30999	76325 (21.53)	8310 (13.44)	84635 (20.33)		
31000–51999	94991 (26.80)	13524 (21.88)	108515 (26.07)		
52000–100000	88934 (25.09)	16982 (27.47)	105916 (25.44)		
>100000	73561 (20.75)	21159 (34.22)	94720 (22.75)		
Education [ *n* (%)]				<0.0001	
Less than high school	63501 (15.57)	19724 (26.70)	83225 (17.28)		
High school or equivalent	157572 (38.65)	28889 (39.11)	186461 (38.72)		
College or above	186640 (45.78)	25258 (34.19)	211898 (44.00)		
Employment [ *n* (%)]	379689 (92.47)	65507 (87.64)	445196 (91.72)	<0.0001	
Adequate exercise [ *n* (%)]	96251 (20.20)	16197 (3.40)	112448 (23.60)	<0.0001	
Smoking status [ *n* (%)]				<0.0001	
Never	231346 (56.32)	34365 (46.07)	265711 (54.75)		
Previous	139230 (33.90)	29182 (39.13)	168412 (34.70)		
Current	40185 (9.78)	11038 (14.80)	51223 (10.55)		
Drinking status [ *n* (%)]				<0.0001	
Never	17168 (4.17)	4378 (5.84)	21546 (4.43)		
Previous	12963 (3.15)	4537 (6.06)	17500 (3.59)		
Current	381591 (92.68)	65981 (88.10)	447572 (91.98)		
The categorical variables are presented as counts (percentage) and compared with the *χ* ^2^ test between the two groups. The sum of the number of participants in all categories is not equal to the total number because of the missing data. Abbreviation: TDI, Townsend deprivation index.					

There were 75209 (15.42%) cases diagnosed with infectious diseases between 2006 and 2010, among whom 2663 (3.54%) suffered from respiratory infectious diseases, 18678 (24.83%) suffered from digestive infectious diseases, and 1051 (1.40%) suffered from blood or sexually transmitted infectious diseases. Furthermore, there were additional infectious diseases that fell outside these three subgroups, such as bacterial infections. For infectious diseases, significant differences were observed in ethnicity, sex, age, TDI, BMI, income, education, working status, smoking status, and drinking status (all *P* < 0.0001) between infected and non-infected individuals.

The participants' characteristics with the lowest and highest consumption of the 13 main food groups are shown in ***Supplementary Table 4*** (available online). The participants who consumed the highest amounts of red and processed meat tended to be current smokers and drinkers, and had a higher BMI than those who consumed the lowest amounts. However, those with the lowest intake of fruits and vegetables were more likely to be current smokers.

### Associations between food groups and infectious diseases

**Figure 2:**
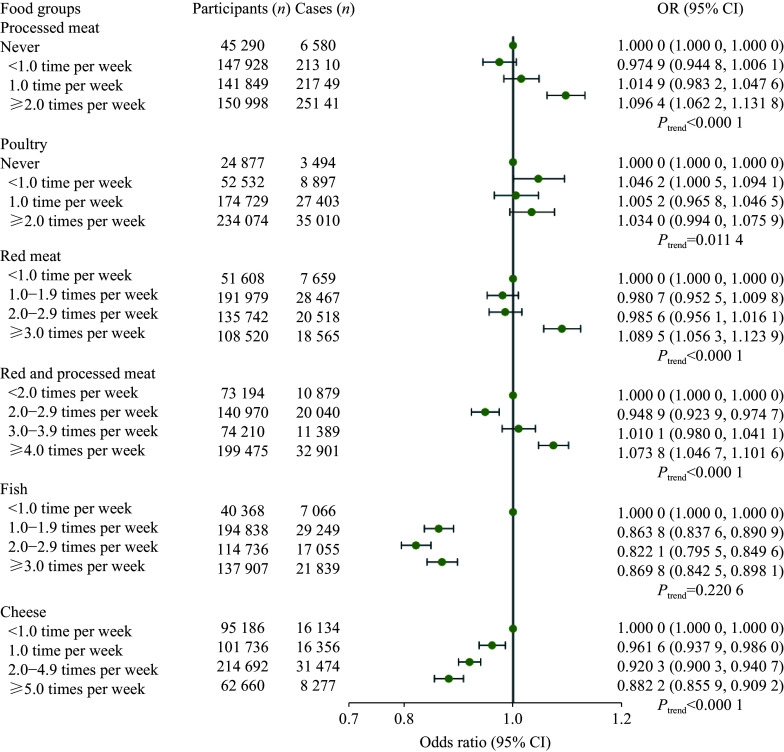
Odds ratio (95% confidence interval) for the associations between animal food intake and infectious diseases in the UK Biobank. Multivariable logistic regression analysis was used and adjusted for age, sex, ethnicity, assessment center, activity, Townsend deprivation index, and education.

**Figure 3:**
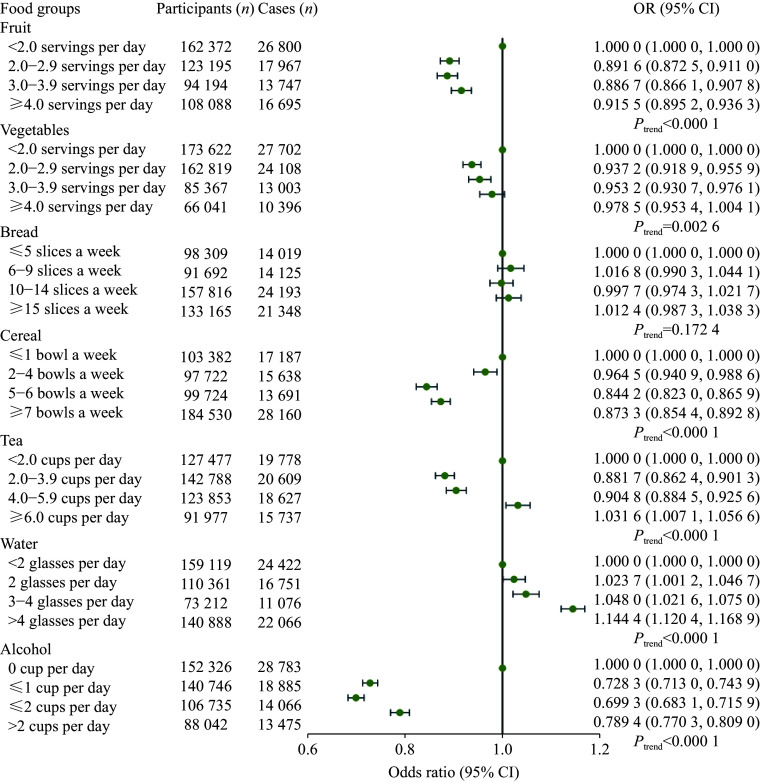
Odds ratio (95% confidence interval) for the associations of other foods with infectious diseases in the UK Biobank. These other foods included plant foods, staple foods, and drinks. Multivariable logistic regression analysis was used, and adjusted for age, sex, ethnicity, assessment center, activity, Townsend deprivation index, and education.

The multivariable associations of the intake of animal and other foods with infectious diseases, adjusted for age, sex, ethnicity, assessment center, activity, TDI, and education, are shown in ***Fig. 2*** and ***Fig. 3***, respectively. For animal foods, the participants with the highest reported intake frequency of processed meat (OR = 1.0964, 95% CI: 1.0622–1.1318), poultry (OR = 1.0340, 95% CI: 0.9940–1.0759), red meat (OR = 1.0895, 95% CI: 1.0563–1.1239), and red and processed meat (OR = 1.0738, 95% CI: 1.0467–1.1016) had a higher risk of infectious diseases than those with the lowest intake frequency. Meanwhile, moderate consumption of red and processed meat (2.0–2.9 times per week; OR = 0.9489, 95% CI: 0.9239–0.9747) reduced the incidence of infectious diseases, compared with those with the lowest consumption. For fish, participants with a higher reported intake frequency had a lower risk of infectious diseases, with all the three ORs less than one. In addition, a higher frequency of cheese intake ( *P*_trend_ < 0.0001) was associated with a lower risk of infectious diseases ( ***Fig. 2*** and ***Supplementary Table 5*** [available online]).

When further determining the associations of different types of fish and red meat (as continuous variables) with infectious diseases, we found that the intake of oily fish was negatively correlated with infectious diseases (OR = 0.9865, 95% CI: 0.9788–0.9943), while a higher intake of non-oily fish may be associated with a higher risk of infectious diseases (OR = 1.0101, 95% CI: 1.0014–1.0188). Beef (OR =1.0347, 95% CI: 1.0251–1.0444), mutton (OR =1.0614, 95% CI: 1.0465–1.0765), and pork (OR =1.0580, 95% CI: 1.0437–1.0723) consumption were all positively associated with overall infection ( ***Supplementary Table 6*** [available online]).

For other foods, participants with a higher intake of fruits and cereals ( *P*_trend_ < 0.0001) had a lower risk of infectious diseases, compared with those with the lowest intake frequency ( ***Fig. 3***). For bread intake, compared with participants who consumed white bread, those who consumed brown bread (OR = 0.8710, 95% CI: 0.8474–0.8952) and whole meal (or whole grain) bread (OR = 0.7752, 95% CI: 0.7602–0.7905) had a lower risk of infectious diseases ( ***Supplementary Table 7*** [available online]). For tea, those who drank 2.0–3.9 cups per day had a lower risk of infection (OR = 0.8817, 95% CI: 0.8624–0.9013), compared with those with the lowest consumption. However, those drinking more than four glasses of water per day had a higher risk of infection than those drinking less than two glasses daily (OR = 1.1444, 95% CI: 1.1204–1.1689). Interestingly, we found that consuming alcohol more frequently than once a week was negatively associated with infectious diseases, with the lowest risk at 3–4 times per week (OR = 0.6858, 95% CI: 0.6699–0.7019) ( ***Supplementary Table 7 ***).

Furthermore, we also evaluated the above associations in three common types of infectious disease subgroups and obtained results of digestive infectious diseases consistent with the main analysis in terms of the effects of processed meat, red meat, fish, cheese, fruit, vegetables, and alcohol consumption ( ***Supplementary Table 8*** [available online]).

### Sensitivity analysis

First of all, compared with those who had not changed their dietary habits in the past five years, the participants with dietary changes because of illness had a higher risk of infectious diseases (OR = 2.109, 95% CI: 2.0612–2.1577), which largely remained significant across three infectious disease subgroups ( ***Supplementary Table 9*** [available online]). Therefore, we further reevaluated the associations between these food groups and infectious diseases by excluding the participants who made a major dietary change because of illness in the past five years, and found that in terms of most food groups, the results derived from the retained 432531 participants were largely similar to the main analysis ( ***Supplementary Table 5 ***).

Second, because one of the most common confounders in epidemiology, sex made a significant difference between infected and non-infected individuals, we conducted a stratified analysis by sex ( ***Table 2***). The results for processed meat, red meat, fish, fruit, tea, and alcohol in 223312 males and 264537 females were broadly consistent with the main analysis. However, there was heterogeneity by sex for the associations between infectious diseases and poultry ( *P*_heterogeneity_ = 0.0168), red meat ( *P*_heterogeneity_ = 0.0058), red and processed meat ( *P*_heterogeneity_ = 0.0078), fish ( *P*_heterogeneity_ = 0.0356), and fruits ( *P*_heterogeneity_ = 0.0021). For example, in terms of poultry, there was a positive association observed in males (more than 2.0 times per week, OR = 1.0758, 95% CI: 1.0094–1.1475) and no association in females (OR = 1.0249, 95% CI: 0.9748–1.0781).

**Table 2:** Associations between dietary factors and infectious diseases in men and women in the UK Biobank

Reported consumptions	Women ( *N*=264537)					Men ( *N*=223312)				*P*_heterogeneity by sex_
	*No*	*no*	OR (95% CI) ^a^	*P*		*No*	*no*	OR (95% CI) ^a^	*P*	
Processed meat										
Never	33246	4699	1.0000 (Ref)			12044	1881	1.0000 (Ref)		
<1.0 time per week	100385	13897	0.9748 (0.9391, 1.0120)	0.1804		47543	7413	0.9864 (0.9308, 1.0457)	0.6445	
1.0 time per week	75620	10927	1.0047 (0.9665, 1.0446)	0.8125		66229	10822	1.0435 (0.9863, 1.1046)	0.1406	
≥2.0 times per week	54385	8363	1.0728 (1.0301, 1.1174)	0.0007		96613	16778	1.1341 (1.0734, 1.1989)	<0.0001	
			*P*_trend_<0.0001					*P*_trend_<0.0001		0.1615
Poultry										
Never	33246	2154	1.0000 (Ref)			8621	1340	1.0000 (Ref)		
<1.0 time per week	100385	4183	1.0289 (0.9704, 1.0913)	0.3412		25317	4714	1.0876 (1.0142, 1.1670)	0.0189	
1.0 time per week	75620	13352	0.9866 (0.9373, 1.0388)	0.6057		82514	14051	1.0525 (0.9869, 1.1233)	0.1212	
≥2.0 times per week	54385	18218	1.0249 (0.9748, 1.0781)	0.3380		105984	16792	1.0758 (1.0094, 1.1475)	0.0255	
			*P*_trend_=0.0143					*P*_trend_=0.0635		0.0168
Red meat										
<1.0 time per week	34324	2154	1.0000 (Ref)			17284	2802	1.0000 (Ref)		
1.0–1.9 times per week	107793	4183	0.9736 (0.9385, 1.0102)	0.1548		84186	13346	1.0080 (0.9609, 1.0578)	0.7446	
2.0–2.9 times per week	70910	13352	0.9785 (0.9410, 1.0176)	0.2762		64832	10433	1.0146 (0.9660, 1.0660)	0.5637	
≥3.0 times per week	51510	18218	1.0557 (1.0136, 1.0998)	0.0092		57010	10541	1.1427 (1.0880, 1.2006)	<0.0001	
			*P*_trend_<0.0001					*P*_trend_<0.0001		0.0058
Red and processed meat										
< 2.0 times per week	51828	7435	1.0000 (Ref)			21366	3444	1.0000 (Ref)		
2.0–2.9 times per week	88126	12004	0.9459 (0.9154, 0.9775)	0.0009		52844	8036	0.9705 (0.9263, 1.0171)	0.2100	
3.0–3.9 times per week	42290	6152	1.0070 (0.9692, 1.0463)	0.7208		31920	5237	1.0359 (0.9851, 1.0894)	0.1694	
≥4.0 times per week	82293	12496	1.0441 (1.0105, 1.0789)	0.0098		117182	20405	1.1232 (1.0764, 1.1723)	<0.0001	
			*P*_trend_<0.0001					*P*_trend_<0.0001		0.0078
Fish										
<1.0 time per week	21731	3601	1.0000 (Ref)			18637	3465	1.0000 (Ref)		
1.0–1.9 times per week	101708	14274	0.8595 (0.8235, 0.8973)	<0.0001		93130	14975	0.8689 (0.8313, 0.9084)	<0.0001	
2.0–2.9 times per week	64009	8733	0.8052 (0.7694, 0.8429)	<0.0001		50727	8322	0.8421 (0.8031, 0.8832)	<0.0001	
≥3.0 times per week	77089	11479	0.8703 (0.8327, 0.9098)	<0.0001		60818	10360	0.8715 (0.8322, 0.9129)	<0.0001	
			*P*_trend_=0.6259					*P*_trend_=0.2869		0.0356

**Table 2:** Associations between dietary factors and infectious diseases in men and women in the UK Biobank (Continued)

Reported consumptions	Women ( *N*=264537)					Men ( *N*=223312)				*P*_heterogeneity by sex_
	*No*	*no*	OR (95% CI) ^a^	*P*		*No*	*no*	OR (95% CI) ^a^	*P*	
Cheese										
<1.0 time per week	57308	9229	1.0000 (Ref)			37878	6905	1.0000 (Ref)		
1.0 time per week	56721	8558	0.9558 (0.9243, 0.9884)	0.0082		45015	7798	0.9700 (0.9341, 1.0072)	0.1127	
2.0–4.9 times per week	110891	14866	0.8908 (0.8648, 0.9177)	<0.0001		103801	16608	0.9524 (0.9217, 0.9842)	0.0036	
≥5.0 times per week	32058	3910	0.8609 (0.8253, 0.8978)	<0.0001		30602	4367	0.9068 (0.8680, 0.9472)	<0.0001	
			*P*_trend_<0.0001					*P*_trend_<0.0001		0.0590
Fruit										
<2.0 servings per day	71977	11110	1.0000 (Ref)			90395	15690	1.0000 (Ref)		
2.0–2.9 servings per day	68054	9307	0.8784 (0.8514, 0.9062)	<0.0001		55141	8660	0.9029 (0.8761, 0.9304)	<0.0001	
3.0–3.9 servings per day	57402	7941	0.8754 (0.8473, 0.9045)	<0.0001		36792	5806	0.8995 (0.8690, 0.9309)	<0.0001	
≥4.0 servings per day	67104	9729	0.8908 (0.8633, 0.9192)	<0.0001		40984	6966	0.9528 (0.9222, 0.9844)	0.0037	
			*P*_trend_<0.0001					*P*_trend_=0.0002		0.0021
Vegetables										
<2.0 servings per day	82317	12433	1.0000 (Ref)			91305	15269	1.0000 (Ref)		
2.0–2.9 servings per day	92359	12823	0.9267 (0.9012, 0.9530)	<0.0001		70460	11285	0.9433 (0.9173, 0.9700)	<0.0001	
3.0–3.9 servings per day	51272	7251	0.9388 (0.9086, 0.9700)	0.0002		34095	5752	0.9620 (0.9289, 0.9961)	0.0296	
≥4.0 servings per day	38589	5580	0.9559 (0.9221, 0.9908)	0.0138		27452	4816	0.9951 (0.9582, 1.0331)	0.7967	
			*P*_trend_=0.0015					*P*_trend_=0.1373		0.1540
Bread										
≤5 slices a week	73017	9904	1.0000 (Ref)			25292	4115	1.0000 (Ref)		
6–9 slices a week	58634	8556	1.0314 (0.9984, 1.0653)	0.0621		33058	5569	0.9930 (0.9482, 1.0399)	0.7646	
10–14 slices a week	86947	12577	1.0204 (0.9906, 1.0510)	0.1818		70869	11616	0.9635 (0.9251, 1.0037)	0.0741	
≥15 slices a week	42392	6340	1.0446 (1.0082, 1.0823)	0.0159		90773	15008	0.9786 (0.9406, 1.0183)	0.2853	
			*P*_trend_=0.0034					*P*_trend_=0.9061		0.4098
Cereal										
≤1 bowl a week	53767	8055	1.0000 (Ref)			49615	9132	1.0000 (Ref)		
2–4 bowls a week	52989	7949	0.9878 (0.9538, 1.0230)	0.4906		44733	7689	0.9450 (0.9124, 0.9787)	0.0016	
5–6 bowls a week	54711	7044	0.8669 (0.8364, 0.8986)	<0.0001		45013	6647	0.8241 (0.7947, 0.8545)	<0.0001	
≥7 bowls a week	101946	14785	0.9148 (0.8868, 0.9436)	<0.0001		82584	13375	0.8372 (0.8114, 0.8638)	<0.0001	
			*P*_trend_<0.0001					*P*_trend_<0.0001		0.0741

**Table 2:** Associations between dietary factors and infectious diseases in men and women in the UK Biobank (Continued)

Reported consumptions	Women ( *N*=264537)					Men ( *N*=223312)				*P*_heterogeneity by sex_
	*No*	*no*	OR (95% CI) ^a^	*P*		*No*	*no*	OR (95% CI) ^a^	*P*	
Tea										
<2.0 cups per day	69331	10129	1.0000 (Ref)			58146	9649	1.0000 (Ref)		
2.0–3.9 cups per day	77238	10449	0.8904 (0.8635, 0.9181)	<0.0001		65550	10160	0.8688 (0.8417, 0.8969)	<0.0001	
4.0–5.9 cups per day	68141	9597	0.9237 (0.8951, 0.9531)	<0.0001		55712	9030	0.8798 (0.8513, 0.9092)	<0.0001	
≥6.0 cups per day	48901	7680	1.0359 (1.0017, 1.0712)	0.0396		43076	8057	1.0205 (0.9860, 1.0562)	0.2463	
			*P*_trend_<0.0001					*P*_trend_=0.0002		0.5456
Water										
<2 glasses per day	73120	10240	1.0000 (Ref)			85999	14182	1.0000 (Ref)		
2 glasses per day	58517	8246	1.0230 (0.9904, 1.0567)	0.1687		51844	8505	1.0297 (0.9986, 1.0618)	0.0609	
3–4 glasses per day	42920	6084	1.0351 (0.9989, 1.0725)	0.0576		30292	4992	1.0721 (1.0332, 1.1123)	0.0002	
>4 glasses per day	87821	13101	1.1314 (1.0986, 1.1651)	<0.0001		53067	8965	1.1725 (1.1366, 1.2095)	<0.0001	
			*P*_trend_<0.0001					*P*_trend_<0.0001		0.8144
Alcohol										
0 cup per day	100879	18001	1.0000 (Ref)			51447	10782	1.0000 (Ref)		
≤1 cup per day	87301	10863	0.7292 (0.7096, 0.7494)	<0.0001		53445	8022	0.7183 (0.6942, 0.7431)	<0.0001	
≤2 cups per day	50622	5897	0.6980 (0.6753, 0.7215)	<0.0001		56113	8169	0.6892 (0.6663, 0.7128)	<0.0001	
>2 cups per day	25735	3326	0.7899 (0.7579, 0.8231)	<0.0001		62307	10149	0.7772 (0.7526, 0.8025)	<0.0001	
			*P*_trend_<0.0001					*P*_trend_=0.9704		0.6160
^a^Adjusted for age, sex, ethnicity, assessment center, activity, Townsend deprivation index, and education. The unequal numbers of groups and each sex are because of the missing data. Abbreviations: *No*, number of participants; *no*, number of cases; CI, confidence interval; OR, odds ratio.										

Third, considering that dietary habits vary across different races, we also estimated the associations between food groups and infectious diseases in different ethnicity or race, and found that the results for the white ethnicity or race were similar to the main analysis, while those for other ethnicities or races showed quite different results ( ***Supplementary Table 10*** [available online]). These results added the evidence to the effects of food groups on the incidence of infectious diseases.

### Associations of diet groups on infectious diseases and the mediators

**Figure 4:**
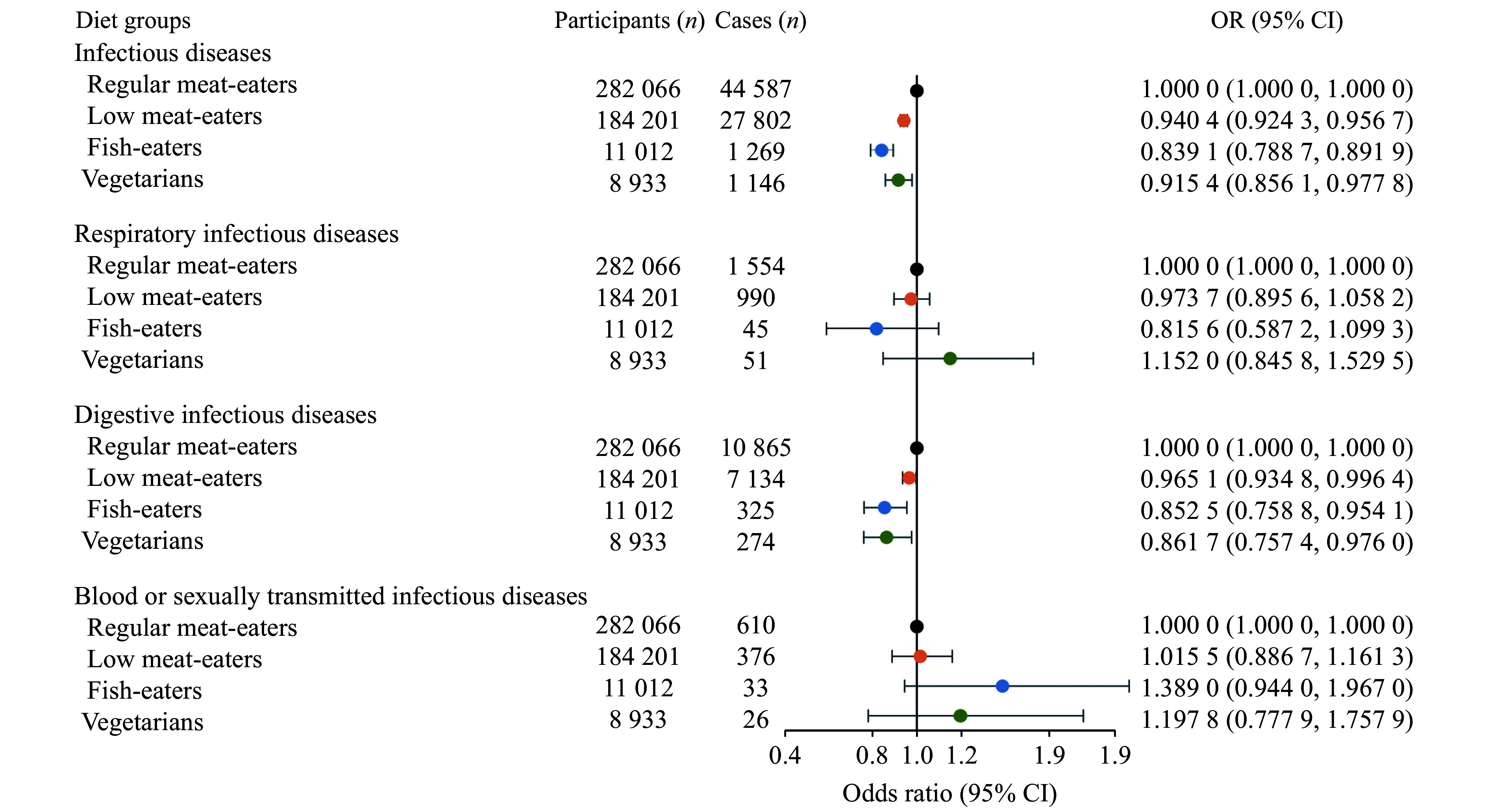
Associations of four diet groups with infectious diseases and three infectious disease subgroups. A multivariable logistic regression analysis was used, and adjusted for age, sex, ethnicity, assessment center, activity, Townsend deprivation index, and education.

The current study defined 282066 regular meat-eaters, 184201 low meat-eaters, 11012 fish-eaters, and 8933 vegetarians. Compared with regular meat eaters, low meat-eaters (OR = 0.9404, 95% CI:0.9243–0.9567), fish-eaters (OR = 0.8391, 95% CI:0.7887–0.8919), and vegetarians (OR = 0.9154, 95% CI: 0.8561–0.9778) had a lower risk of infectious diseases ( ***Fig. 4*** and ***Supplementary Table 11*** [available online]). For the three infectious disease subgroups, the protective effects of the low meat-eaters and fish-eaters were only replicated in digestive infectious diseases.

**Figure 5:**
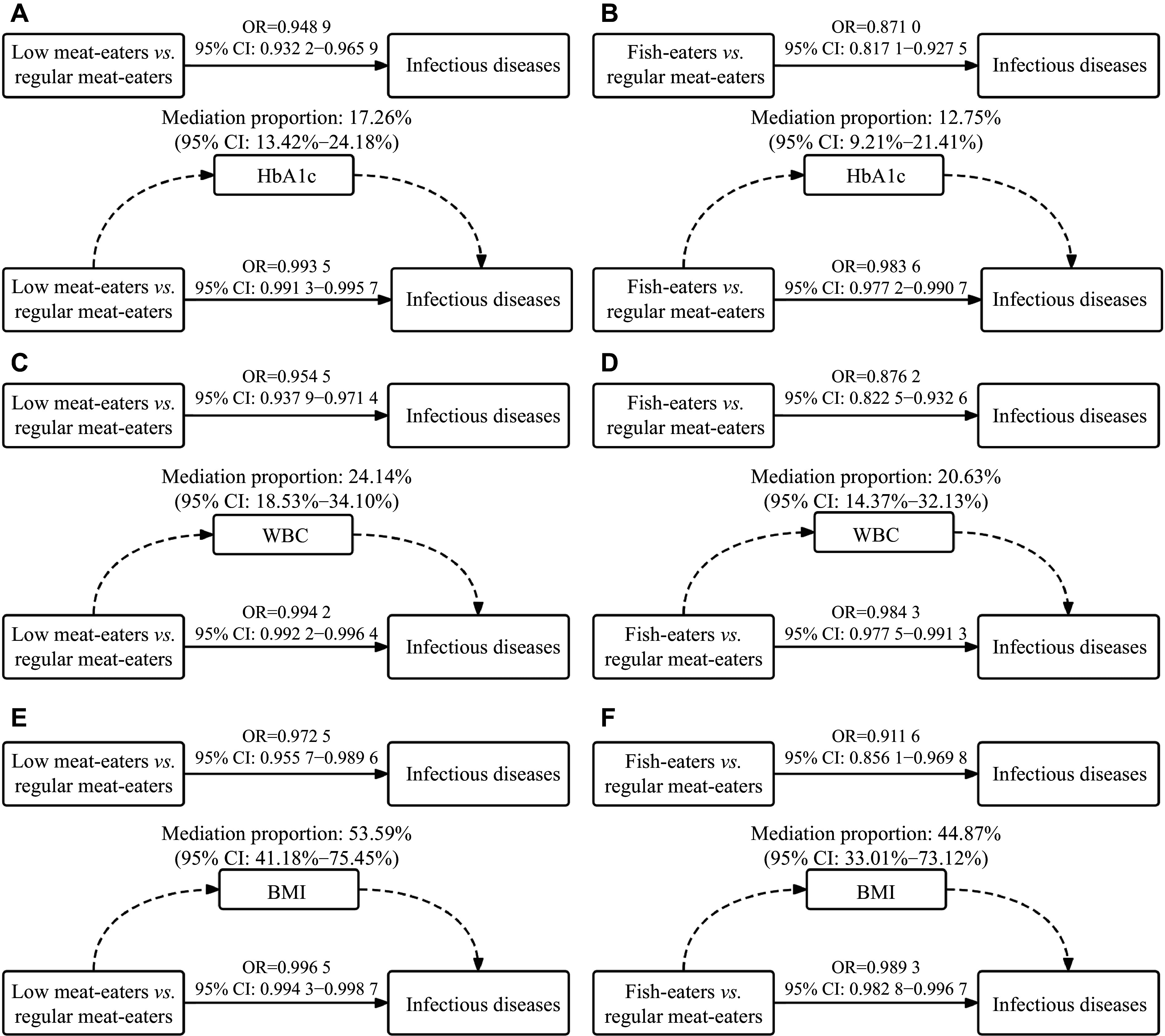
Results of mediation analysis. Mediation effects of low meat-eaters and fish-eaters, compared with regular meat-eaters on infectious diseases by HbA1c (A and B), WBC (C and D), and BMI (E and F). Multivariable logistic regression analysis was used, and adjusted for age, sex, ethnicity, assessment center, activity, Townsend deprivation index, and education. Abbreviations: HbA1c, glycosylated hemoglobin; WBC, white blood cells; BMI, body mass index.

In the mediation analysis, we disclosed the mediation effects of HbA1c and other indicators in the associations observed above. When the mediator was additionally adjusted, the associations were retained ( ***Table 3***). For low meat-eaters and fish-eaters, HbA1c mediated a proportion of 17.26% (95% CI: 13.42%–24.18%) and 12.75% (95% CI: 9.21%–21.41%); WBCs mediated a proportion of 24.14% (95% CI: 18.53%–34.1%) and 20.63% (95% CI: 14.37%–32.13%); while BMI mediated a proportion of 53.59% (95% CI: 41.18%–75.45%) and 44.87% (95% CI: 33.01%–73.12%) in these associations, respectively ( ***Fig. 5***, ***Table 3***, and ***Supplementary Table 12*** [available online]). TG and indicators related to obesity and body fat distribution mediated the associations of low meat-eaters and fish-eaters (with regular meat-eaters as reference) on infectious diseases.

**Table 3:** Mediation effects of diet groups on infectious diseases by potential variables in the UK Biobank

Mediators	Exposure	Effect with mediator adjusted (OR with 95% CI) ^a^	Direct effect (OR with 95% CI) ^a^	Mediation proportion (%) (95% CI) ^a^	*P*
TG	Low meat-eaters *vs.* Ref.	0.9502 (0.9335, 0.9671)	0.9936 (0.9914, 0.9958)	14.19 (10.43, 20.61)	<0.000 1
	Fish-eaters *vs.* Ref.	0.8611 (0.8081, 0.9168)	0.9824 (0.9754, 0.9895)	7.92 (5.41, 12.68)	<0.0001
Glucose	Low meat-eaters *vs.* Ref.	0.9498 (0.9324, 0.9676)	0.9936 (0.9912, 0.9958)	7.27 (5.21, 10.99)	<0.0001
	Fish-eaters *vs.* Ref.	0.846 2 (0.791 3, 0.904 0)	0.980 4 (0.973 7, 0.987 9)	5.37 (3.63, 8.70)	<0.000 1
HbA1c	Low meat-eaters *vs.* Ref.	0.948 9 (0.932 2, 0.965 9)	0.993 5 (0.991 3, 0.995 7)	17.26 (13.42, 24.18)	<0.000 1
	Fish-eaters *vs.* Ref.	0.871 0 (0.817 1, 0.927 5)	0.983 6 (0.977 2, 0.990 7)	12.75 (9.21, 21.41)	<0.000 1
WBC	Low meat-eaters *vs.* Ref.	0.954 5 (0.937 9, 0.971 4)	0.994 2 (0.992 2, 0.996 4)	24.14 (18.53, 34.1)	<0.000 1
	Fish-eaters *vs.* Ref.	0.876 2 (0.822 5, 0.932 6)	0.984 3 (0.977 5, 0.991 3)	20.63 (14.37, 32.13)	<0.000 1
BMI	Low meat-eaters *vs.* Ref.	0.972 5 (0.955 7, 0.989 6)	0.996 5 (0.994 3, 0.998 7)	53.59 (41.18, 75.45)	<0.000 1
	Fish-eaters *vs.* Ref.	0.911 6 (0.856 1, 0.969 8)	0.989 3 (0.982 8, 0.996 7)	44.87 (33.01, 73.12)	<0.000 1
WHR	Low meat-eaters *vs.* Ref.	0.970 3 (0.953 6, 0.987 3)	0.996 3 (0.994 3, 0.998 4)	50.41 (39.72, 70.67)	<0.000 1
	Fish-eaters *vs.* Ref.	0.900 9 (0.846 3, 0.958 3)	0.987 7 (0.980 1, 0.995 1)	36.57 (25.73, 59.84)	<0.000 1
Body fat percentage	Low meat-eaters *vs.* Ref.	0.968 1 (0.951 2, 0.985 2)	0.996 0 (0.994 0, 0.998 1)	49.26 (38.37, 67.43)	<0.000 1
	Fish-eaters *vs.* Ref.	0.917 5 (0.861 3, 0.976 5)	0.989 9 (0.982 4, 0.997 1)	47.39 (33.09, 77.04)	<0.000 1
Whole body fat mass	Low meat-eaters *vs.* Ref.	0.971 7 (0.954 8, 0.989 0)	0.996 5 (0.994 3, 0.998 7)	53.45 (41.84, 76.09)	<0.000 1
	Fish-eaters *vs.* Ref.	0.919 1 (0.862 8, 0.978 3)	0.990 0 (0.983 2, 0.997 5)	46.87 (33.30, 77.84)	<0.000 1
Arms fat mass	Low meat-eaters *vs.* Ref.	0.964 5 (0.948 0, 0.981 4)	0.995 5 (0.993 3, 0.997 6)	39.69 (30.44, 55.78)	<0.000 1
	Fish-eaters *vs.* Ref.	0.893 2 (0.839 2, 0.949 9)	0.986 6 (0.979 5, 0.993 8)	31.54 (22.82, 51.12)	<0.000 1
Leg fat mass	Low meat-eaters *vs.* Ref.	0.961 4 (0.944 9, 0.978 2)	0.995 2 (0.993 1, 0.997 4)	34.99 (26.95, 51.17)	<0.000 1
	Fish-eaters *vs.* Ref.	0.887 9 (0.834 2, 0.944 2)	0.986 1 (0.979 3, 0.993 4)	28.44 (20.15, 47.18)	<0.000 1
Trunk fat mass	Low meat-eaters *vs.* Ref.	0.969 1 (0.952 2, 0.986 3)	0.996 1 (0.993 9, 0.998 2)	49.49 (38.15, 68.13)	<0.000 1
	Fish-eaters *vs.* Ref.	0.910 7 (0.855 0, 0.969 2)	0.989 3 (0.982 3, 0.995 9)	42.58 (30.17, 66.34)	<0.000 1
^a^Adjusted for age, sex, ethnicity, assessment center, activity, Townsend deprivation index, and education.Abbreviations: CI, confidence interval; OR, odds ratio; TG, total cholesterol; WBC, white blood cells; BMI, body mass index; WHR, waist-to-hip ratio.					

### Discussion

In this large-scale contemporary study, we conducted comprehensive analyses to investigate the associations between diet and infectious diseases, and found that the consumption frequency of different food groups was associated with the risk of infection. A series of sensitivity analyses were performed to confirm the main results. Diet groups were also associated with the risk of infectious diseases, and HbA1c, WBC, and BMI may mediate the observed significant associations.

Investigators have been unanimously concerned about the health effects of meat consumption ^[ 24, 26] ^. Nowadays, people pay increasing attention to a balanced diet. The extensive consumption of meat may replace the consumption of other foods, such as vegetables, and result in changes to meal composition. In addition, Europeans have a habit of consuming raw meat like medium-rare steak, and the increased frequency of raw meat intake increases exposure to pathogens, contributing to the high risk of digestive infectious diseases ^[ 27] ^. These findings are consistent with the current study that the higher meat consumption may be associated with a higher risk of digestive infectious diseases. Furthermore, our results showed that moderate meat consumption reduced the risk of infection, and we suspect that the reason may be because of the fact that eating meat in small amounts helps the body replenish nutrients like protein, lipids, and minerals ^[ 28] ^. Fish, as one of the most important sources of omega-3 polyunsaturated fatty acids, is associated with various health benefits. A meta-analysis of prospective cohort studies indicated that higher fish consumption might reduce all-cause mortality ^[ 29– 30] ^. Our results showed that a higher intake of fish, especially oily fish, was associated with a lower risk of infectious diseases, providing further evidence that fish intake has health benefits. Cheese, a fresh or matured food obtained from the coagulation of milk, is rich in nutritional components, such as proteins, bioactive proteins, amino acids, minerals, and vitamins ^[ 31] ^. In the current study, we found that a higher frequency of cheese consumption was positively associated with a lower risk of infectious diseases, in both women and men. Consuming cheese more than five times a week may be recommended to reduce the infectious risk based on the current results. Nevertheless, this may also be attributed to the common practice of consuming cheese with fresh foods.

Dietary guidelines worldwide recommend consuming more fruits and vegetables, which are rich in vitamins, minerals, dietary fiber, and a variety of phytochemicals to maintain health ^[ 32] ^. In the current study, we separately investigated the associations of fruits and vegetables with infectious diseases, and found that a daily intake of 3.0–3.9 servings of fruit and 2.0–2.9 servings of vegetables was associated with a lower risk of infectious diseases. These findings are consistent with a meta-analysis of 26 cohort studies, which found that five or more servings of fruits and vegetables per day were associated with less damage to health ^[ 33] ^.

Bread and cereal are staple foods of Europeans. Whole grain bread offers advantages over other types of bread, which may be related to the ingredients, processing technology, and edible methods ^[ 34– 35] ^. Tea, as a pleasant, popular, and socially accepted drink, is the richest source of a class of antioxidants called flavonoids and has the function of preventing intestinal flora infection and enhancing body immunity ^[ 36] ^. Combining the above evidence and our findings, moderate drinking of tea (2.0–3.9 cups daily) is worth advocating, but excessive tea intake increases disease risk, which may be related to the inhibition of essential trace elements.

An intriguing trend was observed in the current study, where individuals consuming more than four glasses of water per day exhibited a higher risk of infection than those consuming fewer than two glasses. Based on the literature, we posited the following potential reasons: although in developed countries, there were millions of cases of waterborne infections each year ^[ 37– 38] ^. Water intake is associated with the opportunity for exposure to pathogens in drinking water, where there may be over 500 types of waterborne pathogens. Despite strict management of drinking water quality being accessible in developed countries, it may not screen for fungi, algae, protozoa, and less significant bacteria, which leads to pathogens or organisms entering households through water as a medium of transmission. Additionally, the aging of drinking water treatment or distribution systems may also result in pathogen invasion ^[ 39– 40] ^. Furthermore, areas with poor water quality often coincide with dense populations, lower economies, and other social factors, which may be associated with respiratory infections, sexually transmitted diseases, and other issues ^[ 38] ^.

In general, after being ill, changing dietary habits is the common non-drug treatment to adjust the body status or prevent the disease progression; however, it may lead to a selection bias of participants ^[ 41] ^. The current study took this bias into consideration by excluding those participants who reported having made major changes in the past five years because of illness, and the results were consistent with those before the exclusion, indicating that the associations between diet and infectious diseases were valid.

We observed a sex-based heterogeneity in the associations of poultry, red meat, red and processed meat, fish, and fruit with infectious diseases. While these differences may not be clearly explained now, they indicate that the effect of sex on infectious diseases should not be ignored. There is evidence that the pathogen-induced immune responses are significantly influenced by the host's sex in many instances, and several genes have been observed to affect susceptibility to viruses, bacteria, parasites, fungi, and worms ^[ 42] ^.

Ethnicity is another important social determinant of infectious diseases besides sex ^[ 43] ^. Our findings of the white ethnicity were aligned with the main results, while there were fewer associations in the Asian or African group. Such differences may be linked to genetic factors and immune responses, or it may have been difficult to detect those associations in a limited sample size.

Compared with the regular meat-eaters, the low meat-eaters, fish-eaters, and vegetarians had a lower risk of infectious diseases, suggesting that dietary preferences of meat and fish indeed influence the infection risk. Further analysis disclosed the mediation effects of nutritional status-related indicators such as BMI/BFP, metabolic-related indicators such as HbA1c, and immune-related indicators such as WBC in the above-mentioned associations. According to the findings, we speculate that several aspects mentioned above may be potential factors or pathways influencing the risk of infectious diseases because of dietary influences. Among these mediators, the intermediate effect values of nutrition-related indicators are relatively significant. This suggests that it may be possible to reduce the risk of infectious diseases by adjusting the overall nutritional status of the population through the establishment of healthy dietary habits.

Nonetheless, the current study has several limitations. First, although we defined the dietary factors using the baseline survey data to help us confirm the intake of food groups, the eating habits of participants may change before suffering from infectious diseases, which may lead to inaccurately estimated associations between diet and infections. Second, the dietary data coming from the questionnaire was self-reported, which may have produced measurement errors and bias. Third, the current study focused on the frequency of food groups, and because of the lack of uniform standards for quantifying, the estimated associations between diet and infectious diseases may be biased. Fourth, because of variations in recurrence rates among different infectious diseases and the lack of practical significance in aggregating disease frequencies, the current study did not analyze the specific associations between diet and each infectious disease individually. However, future exploration and validation of such associations through alternative approaches, such as animal experiments, may be necessary.

## SUPPLEMENTARY DATA

Supplementary data to this article can be found online.
