# Supplementary material for: Associations of diet with infectious diseases in UK Biobank
Source: J Biomed Res. 2024 May 29;38(6):597–612. doi: 10.7555/JBR.37.20230319 (PMC11629163; doi:10.7555/JBR.37.20230319)
Supplement: Supplementary file 1 — Supplementary data to this article can be found online. [file jbr-38-6-597-S1.pdf]

Available online at www.jbr-pub.org.cn

Open Access at PubMed Central

Journal of Biomedical Research, 2024 38(6): 1–15

Supplementary Data

JBR

Associations of diet with infectious diseases in UK Biobank

Junlan Tu[1,△], Xuehong Cai[1,△], Yifan Wang[2,△], Xiangyu Ye[1], Meijie Yu[1], Sheng Yang[3], Rongbin Yu[1,✉], Peng Huang[1,✉]

[1]*Department of Epidemiology, National Vaccine Innovation Platform, Center for Global Health, School of Public Health, Nanjing Medical University, Nanjing, Jiangsu 211166, China;*
[2]*Department of Infectious Disease, Jurong Hospital Affiliated to Jiangsu University, Jurong, Jiangsu 212400, China;*
[3]*Department of Biostatistics, National Vaccine Innovation Platform, Center for Global Health, School of Public Health, Nanjing Medical University, Nanjing, Jiangsu 211166, China.*

Supplementary Table 1 **Definitions of food and food groups from the UK Biobank**

Food groups	Items	UK Biobank field code	Definition	Categories
Animal foods				
Processed meat	–	1349	1) Defined "Do not know" and "Prefer not to answer" as missing;<br>2) Combined the top three frequencies into one category.	1) Never<br>2) <1.0 time per week<br>3) 1.0 time per week<br>4) ≥2.0 times per week
Poultry	–	1359	1) Defined "Do not know" and "Prefer not to answer" as missing;<br>2) Combined the top three frequencies into one category.	1) Never<br>2) <1.0 time per week<br>3) 1.0 time per week<br>4) ≥2.0 times per week
Red meat	Beef<br>Lamb<br>Pork	1369<br>1379<br>1389	1) Defined "Do not know" and "Prefer not to answer" as missing;<br>2) Defined "Never" as 0 times, "Less than once a week" as 0.5 times, "Once a week" as 1 time, "2–4 times a week" as 3 times, "5–6 times a week" as 5.5 times, "Once or more daily" as 7 times per week;<br>3) Summed the frequencies for beef, pork, and lamb/mutton.	1) <1.0 time per week<br>2) 1.0–1.9 times per week<br>3) 2.0–2.9 times per week<br>4) ≥3.0 times per week
Processed and red meat	Processed meats<br>Beef<br>Lamb<br>Pork	1349<br>1369<br>1379<br>1389	1) Defined "Do not know" and "Prefer not to answer" as missing;<br>2) Defined "Never" as 0 times, "Less than once a week" as 0.5 times, "Once a week" as 1 time, "2–4 times a week" as 3 times, "5–6 times a week" as 5.5 times, "Once or more daily" as 7 times per week;<br>3) Summed the frequencies for processed meats, beef, pork, and lamb.	1) <2.0 times per week<br>2) 2.0–2.9 times per week<br>3) 3.0–3.9 times per week<br>4) ≥4.0 times per week

△These authors contributed equally to this work.

✉Corresponding authors: Rongbin Yu and Peng Huang, Department of Epidemiology, Center for Global Health, School of Public Health, Nanjing Medical University, Nanjing, Jiangsu 211166, China. E-mails: rongbinyu@njmu.edu.cn (Yu) and huangpeng@njmu.edu.cn (Huang).

Received: 30 December 2023; Revised: 27 April 2024; Accepted: 06 May 2024; Published online: 29 May 2024

© 2024 by Journal of Biomedical Research.

CLC number: R155.3, Document code: A

The authors reported no conflict of interests.

This is an open access article under the Creative Commons Attribution (CC BY 4.0) license, which permits others to distribute, remix, adapt and build upon this work, for commercial use, provided the original work is properly cited.

https://doi.org/10.7555/JBR.37.20230319

Supplementary Table 1 **Definitions of food and food groups from the UK Biobank** (Continued)

Food groups	Items	UK Biobank field code	Definition	Categories
Animal foods				
Fish	Oily fish<br>Non-oily fish	1329<br>1339	1) Defined "Do not know" and "Prefer not to answer" as missing;<br>2) Defined "Never" as 0 times, "Less than once a week" as 0.5 times, "Once a week" as 1 time, "2–4 times a week" as 3 times, "5–6 times a week" as 5.5 times, "Once or more daily" as 7 times per week;<br>3) Summed the frequencies for oily fish and non-oily fish.	1) <1.0 time per week<br>2) 1.0–1.9 times per week<br>3) 2.0–2.9 times per week<br>4) ≥3.0 times per week
Cheese	–	1408	1) Defined "Do not know" and "Prefer not to answer" as missing;<br>2) Combined the bottom two frequencies into the lowest category;<br>3) Combined the top two frequencies into the highest category.	1) <1.0 time per week<br>2) 1.0 time per week<br>3) 2.0–4.9 times per week<br>4) ≥5.0 times per week
Other foods				
Fruits	Fresh fruit<br>Dried fruit	1309<br>1319	1) Defined "Do not know" and "Prefer not to answer" as missing;<br>2) Defined "Less than one" as 0 servings, and "one piece of fresh fruit" and "two pieces of dried fruit" were counted as a serving;<br>3) Summed the frequencies for fresh fruit and dried fruit.	1) <2.0 servings per day<br>2) 2.0–2.9 servings per day<br>3) 3.0–3.9 servings per day<br>4) ≥4.0 servings per day
Vegetables	Cooked vegetables<br>Salad/raw vegetables	1289<br>1299	1) Defined "Do not know" and "Prefer not to answer" as missing;<br>2) Defined "Less than one" as 0 serving, and "two heaped tablespoons of salad/raw or cooked vegetables" as a serving;<br>3) Summed the frequencies for salad/raw or cooked vegetables.	1) <2.0 servings per day<br>2) 2.0–2.9 servings per day<br>3) 3.0–3.9 servings per day<br>4) ≥4.0 servings per day
Bread	–	1438	Defined "Do not know" and "Prefer not to answer" as missing, and defined "Less than one" as 0 slices.	1) ≤5 slices a week<br>2) 6–9 slices a week<br>3) 10–14 slices a week<br>4) ≥15 slices a week
Cereals	–	1458	Defined "Do not know" and "Prefer not to answer" as missing, and defined "Less than one" as 0 bowls.	1) ≤1 bowl a week<br>2) 2–4 bowls a week<br>3) 5–6 bowls a week<br>4) ≥7 bowls a week
Tea	–	1488	Defined "Do not know" and "Prefer not to answer" as missing, and defined "Less than one" as 0 cups.	1) <2.0 cups per day<br>2) 2.0–3.9 cups per day<br>3) 4.0–5.9 cups per day<br>4) ≥6.0 cups per day
Water	–	1528	Defined "Do not know" and "Prefer not to answer" as missing, and defined "Less than one" as 0 glasses;	1) <2 glasses per day<br>2) 2 glasses per day<br>3) 3–4 glasses per day<br>4) >4 glasses per day
Alcohol	Average weekly red wine intake<br>Average weekly champagne plus white wine intake<br>Average weekly beer plus cider intake<br>Average weekly spirits intake<br>Average weekly fortified wine intake<br>Average weekly intake of other alcoholic drinks	1568<br>1578<br>1588<br>1598<br>1608<br>5364	1) Defined "Do not know" and "Prefer not to answer" as missing;<br>2) Summed their total weekly consumption of alcohol;<br>3) Divided weekly consumption by 7.	1) 0 cups per day<br>2) ≤1 cup per day<br>3) ≤2 cups per day<br>4) >2 cups per day
Alcohol frequency	–	1558	1) Defined "Prefer not to answer" as missing;<br>2) Defined "Never", "Special occasions only" and "One to three times a month" as less than once a week	1) 1 time a week<br>2) 1–2 times a week<br>3) 3–4 times a week<br>4) Daily or almost daily

Supplementary Table 2 **Definitions of whole infectious diseases and three common infectious diseases from the UK Biobank**

Outcome variables	The UK Biobank field code	Definition	Note
Infectious diseases	41202, 41270, 41262, 41280, 41203, 41271, 41263, 41281	ICD-10 codes A00–B99 and J00–J22;<br>ICD-9 codes 001–139 and 480–487	Diagnosed after enrollment
Respiratory infectious diseases		ICD-10 codes A15, A37, A39, B01, B02, B05, B06, B26, and J09–J11;<br>ICD-9 codes 001, 012, 033, 036, 053, 055, 056, 072 and 487	
Digestive infectious diseases		ICD-10 codes A00–A09, B15, B17.2, B67, B68, B77, B80, and B82;<br>ICD-9 codes 001–009, 0701, and 122	
Blood or sexually transmitted infectious diseases		ICD-10 codes A50–A64, B16, B17.1, B18.0, B18.1, B18.2, and B20–B24;<br>ICD-9 codes 0703 and 090–099	

Supplementary Table 3 **Definitions of the variables used in the current study from the UK Biobank**

Variables	The UK Biobank field code	Definition	Note
Genetic sex & Sex	22001, 31	Match the genetic sex and reported sex, then retain the sex-consistent participants:<br>1) male<br>2) female	—
Ethnic background	21000	Grouped into four categories:<br>1) the white<br>2) the Asian or Asian British<br>3) the Black or Black British<br>4) the mixed or others	—
Age when attended assessment center	21003	Grouped into 6 categories (year):<br>1) <45<br>2) 45–49<br>3) 50–54<br>4) 55–59<br>5) 60–65<br>6) ≥65	5 Years as an interval
Townsend deprivation index at recruitment	189	—	—
Body mass index	21001	Grouped into four categories (kg/m^2):<br>1) <18.5<br>2) ≥18.5<br>3) ≥25<br>4) ≥30	World Health Organization standards
Average total household income before tax	738	Grouped into five levels:<br>1) <£18 000;<br>2) £18 000–£30 999;<br>3) £31 000–£51 999;<br>4) £52 000–£;<br>5) > £100 000	Defined "Do not know" and "Prefer not to answer" as missing
Qualifications	6138	Grouped into three categories:<br>1) the lowest<br>2) the middle<br>3) the highest	1) Defined "Prefer not to answer" as missing;<br>2) Defined "College or University degree" and "A levels/AS levels or equivalent" as the highest category;<br>3) Defined "O levels/GCSEs or equivalent", "CSEs or equivalent", "NVQ or HND or HNC or equivalent", and "Other professional qualifications, *e.g.*, nursing, teaching" as the middle category;<br>4) Defined "None of the above" as the lowest category.
Current employment status	6142	Grouped into two categories:<br>1) employed<br>2) unemployed	1) Defined "Prefer not to answer" as missing;<br>2) Defined "In paid employment or self-employed", "Retired", "Doing unpaid or voluntary work", and "Full or part-time student" as the employed;<br>3) Defined "Looking after home and/or family", "Unable to work because of sickness or disability", "Unemployed", and "None of the above" as the unemployed.

Supplementary Table 3 **Definitions of the variables used in the current study from the UK Biobank** (Continued)

Variables	The UK Biobank field code	Definition	Note
Physical activity	884, 894, 904, 914	Grouped into two categories:<br>1) adequate exercise<br>2) lack of exercise	1) Defined "moderate at least five days a week or vigorous once a week" as "Adequate exercise";<br>2) Defined "more than 150 minutes of moderate activity per week" as "Adequate exercise";<br>3) Defined "more than 75 minutes of vigorous activity per week" as "Adequate exercise";<br>4) The others were defined as "Lack of exercise".
Smoking status	20116	Grouped into three categories:<br>1) never smoked<br>2) smoked previously but quit when reported<br>3) keep smoking when reported	—
Alcohol drinker status	20117	Grouped into three categories:<br>1) never drunk<br>2) drunk previously but quit when reported<br>3) keep drinking when reported	—
The UK Biobank assessment centre	54	—	—
Triglycerides (TG)	30870	—	Continuous, mmol/L
Glucose	30740	—	Continuous, mmol/L
Glycosylated hemoglobin (HbA1c)	30750	—	Continuous, mmol/L
Waist-to-hip ratio (WHR)	48, 49	WHR= (Waist circumference) / (Hip circumference)	—
Body fat percentage (BFP)	23099	—	—
Whole body fat mass	23100	—	Continuous, kg
Arm fat mass	23124, 23120	Fat mass of a pair of arms	Continuous, kg
Leg fat mass	23116, 23112	Fat mass of a pair of legs	—
Trunk fat mass	23128	—	—

Supplementary Table 4 (available online) shows the correlation of anthropometric variables with the intake of main food groups in the UK Biobank.

Supplementary Table 5 Associations between the intake frequency of 13 food groups and infectious diseases in the UK Biobank

Reported consumption at recruitment	All participants (N=487 849)			Participants who did not report changing their diet because of illness (N=432 531)		
	Participants (n)	Cases (n)	OR (95% CI)	Participants (n)	Cases (n)	OR (95% CI)
Processed meat						
Never	45 290	6 580	1.000 0 (Ref)	39 517	5074	1.000 0 (Ref)
<1.0 time per week	147 928	21 310	0.974 9 (0.944 8, 1.006 1)	131 993	17 123	0.984 2 (0.950 4, 1.019 4)
1.0 time per week	141 849	21 749	1.014 9 (0.983 2, 1.047 6)	126 427	17 513	1.033 1 (0.997 2, 1.070 3)
⩾2.0 times per week	150 998	25 141	1.096 4 (1.062 2, 1.131 8)	133 728	20 057	1.117 1 (1.078 3, 1.157 5)
			P_{trend}<0.000 1			P_{trend}<0.000 1
Poultry						
Never	24 877	3494	1.000 0 (Ref)	22 252	2767	1.000 0 (Ref)
<1.0 time per week	52 532	8897	1.046 2 (1.000 5, 1.094 1)	46 764	7111	1.054 1 (1.003 5, 1.107 6)
1.0 time per week	174 729	27 403	1.005 2 (0.965 8, 1.046 5)	156 107	22 130	1.019 2 (0.975 1, 1.065 6)
⩾2.0 times per week	234 074	35 010	1.034 0 (0.994 0, 1.075 9)	206 630	27 764	1.045 6 (1.001 0, 1.092 5)
			P_{trend}=0.011 4			P_{trend}=0.007 4
Red meat						
<1 time per week	51 608	7659	1.000 0 (Ref)	44 693	5787	1.000 0 (Ref)
1.0–1.9 times per week	191 979	28 467	0.980 7 (0.952 5, 1.009 8)	170 955	22 843	0.998 7 (0.966 8, 1.031 8)
2.0–2.9 times per week	135 742	20 518	0.985 6 (0.956 1, 1.016 1)	121 346	16 583	1.005 5 (0.972 0, 1.040 2)
⩾3.0 times per week	108 520	18 565	1.089 5 (1.056 3, 1.123 9)	95 537	14 725	1.110 8 (1.073 1, 1.150 0)
			P_{trend}<0.000 1			P_{trend}<0.000 1
Red and processed meat						
<2.0 times per week	73 194	10 879	1.000 0 (Ref)	63 368	8290	1.000 0 (Ref)
2.0–2.9 times per week	140 970	20 040	0.948 9 (0.923 9, 0.974 7)	126 278	16 228	0.960 9 (0.932 8, 0.990 0)
3.0–3.9 times per week	74 210	11 389	1.010 1 (0.980 0, 1.041 1)	66 103	9117	1.018 8 (0.985 2, 1.053 6)
⩾4.0 times per week	199 475	32 901	1.073 8 (1.046 7, 1.101 6)	176 782	26 303	1.091 1 (1.060 5, 1.122 6)
			P_{trend}<0.000 1			P_{trend}<0.000 1
Fish						
<1 time per week	40 368	7066	1.000 0 (Ref)	34 842	5377	1.000 0 (Ref)
1.0–1.9 times per week	194 838	29 249	0.863 8 (0.837 6, 0.890 9)	175 544	23 905	0.870 2 (0.841 1, 0.900 5)
2.0–2.9 times per week	114 736	17 055	0.822 1 (0.795 5, 0.849 6)	102 444	13 745	0.819 0 (0.789 8, 0.849 5)
⩾3.0 times per week	137 907	21 839	0.869 8 (0.842 5, 0.898 1)	119 701	16 911	0.856 0 (0.826 2, 0.887 1)
			P_{trend}=0.220 6			P_{trend}=0.001 2
Cheese						
<1.0 time per week	95 186	16 134	1.000 0 (Ref)	80 990	12 176	1.000 0 (Ref)
1.0 time per week	101 736	16 356	0.961 6 (0.937 9, 0.986 0)	89 751	12 982	0.976 1 (0.949 1, 1.003 8)
2.0–4.9 times per week	214 692	31 474	0.920 3 (0.900 3, 0.940 7)	194 498	25 989	0.940 8 (0.918 0, 0.964 2)
⩾5.0 times per week	62 660	8277	0.882 2 (0.855 9, 0.909 2)	57 828	7014	0.905 8 (0.876 3, 0.936 3)
			P_{trend}<0.000 1			P_{trend}<0.000 1
Fruit						
<2.0 servings per day	162 372	26 800	1.000 0 (Ref)	144 874	21 617	1.000 0 (Ref)
2.0–2.9 servings per day	123 195	17 967	0.891 6 (0.872 5, 0.911 0)	110 028	14 545	0.886 5 (0.865 7, 0.907 7)
3.0–3.9 servings per day	94 194	13 747	0.886 7 (0.866 1, 0.907 8)	83 320	10 872	0.867 6 (0.845 3, 0.890 5)

Supplementary Table 5 Associations between the intake frequency of 13 food groups and infectious diseases in the UK Biobank (Continued)

Reported consumption at recruitment	All participants (N=487 849)			Participants who did not report changing their diet because of illness (N=432 531)		
	Participants (n)	Cases (n)	OR (95% CI)	Participants (n)	Cases (n)	OR (95% CI)
⩾4.0 servings per day	108 088	16 695	0.915 5 (0.895 2, 0.936 3)	94 309	12 904	0.886 4 (0.864 5, 0.908 8)
			P_{trend}<0.000 1			P_{trend}<0.000 1
Vegetables						
<2.0 servings per day	173 622	27 702	1.000 0 (Ref)	153 803	22 008	1.000 0 (Ref)
2.0–2.9 servings per day	162 819	24 108	0.937 2 (0.918 9, 0.955 9)	145 232	19 402	0.933 7 (0.913 6, 0.954 2)
3.0–3.9 servings per day	85 367	13 003	0.953 2 (0.930 7, 0.976 1)	75 580	10 343	0.942 9 (0.918 4, 0.968 0)
⩾4.0 servings per day	66 041	10 396	0.978 5 (0.953 4, 1.004 1)	57 916	8 185	0.969 9 (0.942 4, 0.998 2)
			P_{trend}=0.002 6			P_{trend}=0.000 4
Bread						
⩽5 slices a week	98 309	14 019	1.000 0 (Ref)	87 166	11 168	1.000 0 (Ref)
6–9 slices a week	91 692	14 125	1.016 8 (0.990 3, 1.044 1)	81 795	11 370	1.024 3 (0.994 8, 1.054 6)
10–14 slices a week	157 816	24 193	0.997 7 (0.974 3, 1.021 7)	140 717	19 460	1.006 0 (0.980 0, 1.032 8)
⩾15 slices a week	133 165	21 348	1.012 4 (0.987 3, 1.038 3)	117 737	16 957	1.020 6 (0.992 6, 1.049 5)
			P_{trend}=0.172 4			P_{trend}=0.235 8
Cereal						
⩽1 bowl a week	103 382	17 187	1.000 0 (Ref)	91 971	13 821	1.000 0 (Ref)
2–4 bowls a week	97 722	15 638	0.964 5 (0.940 9, 0.988 6)	86 327	12 352	0.953 3 (0.927 6, 0.979 7)
5–6 bowls a week	99 724	13 691	0.844 2 (0.823 0, 0.865 9)	89 625	11 182	0.846 2 (0.822 9, 0.870 2)
⩾7 bowls a week	184 530	28 160	0.873 3 (0.854 4, 0.892 8)	163 208	22 335	0.856 9 (0.836 4, 0.877 9)
			P_{trend}<0.000 1			P_{trend}<0.000 1
Tea						
<2.0 cups per day	127 477	19 778	1.000 0 (Ref)	113 182	15 773	1.000 0 (Ref)
2.0–3.9 cups per day	142 788	20 609	0.881 7 (0.862 4, 0.901 3)	127 606	16 614	0.890 7 (0.869 3, 0.912 6)
4.0–5.9 cups per day	123 853	18 627	0.904 8 (0.884 5, 0.925 6)	110 142	14 937	0.908 6 (0.886 1, 0.931 6)
⩾6.0 cups per day	91 977	15 737	1.031 6 (1.007 1, 1.056 6)	80 767	12 414	1.031 9 (1.004 8, 1.059 7)
			P_{trend}<0.000 1			P_{trend}<0.000 1
Water						
<2 glasses per day	159 119	24 422	1.000 0 (Ref)	144 285	20 343	1.000 0 (Ref)
2 glasses per day	110 361	16 751	1.023 7 (1.001 2, 1.046 7)	98 545	13 629	1.016 7 (0.992 3, 1.041 6)
3–4 glasses per day	73 212	11 076	1.048 0 (1.021 6, 1.075 0)	64 640	8 781	1.026 0 (0.997 6, 1.055 2)
>4 glasses per day	140 888	22 066	1.144 4 (1.120 4, 1.168 9)	122 088	16 655	1.088 3 (1.063 1, 1.114 2)
			P_{trend}<0.000 1			P_{trend}<0.000 1
Alcohol						
0 cups per day	152 326	28 783	1.000 0 (Ref)	126 497	20 807	1.000 0 (Ref)
⩽1 cup per day	140 746	18 885	0.728 3 (0.713 0, 0.743 9)	127 502	15 703	0.768 3 (0.750 4, 0.786 7)
⩽2 cups per day	106 735	14 066	0.699 3 (0.683 1, 0.715 9)	97 738	11 917	0.749 5 (0.730 4, 0.769 0)
>2 cups per day	88 042	13 475	0.789 4 (0.770 3, 0.809 0)	80 794	11 511	0.855 1 (0.832 3, 0.878 4)
			P_{trend}<0.000 1			P_{trend}=0.147 6

Multivariable logistic regression analysis was used, and adjusted for age, sex, ethnicity, assessment center, BMI, activity, Townsend deprivation index, income, and education. The sum of the number of people in all categories is not equal to the total number because of missing data. Abbreviations: CI, confidence interval; OR, odds ratio.

Supplementary Table 6 **Associations between different types of fish/red meat and infectious diseases (and common infectious diseases subgroups)**

Outcome variables	Food group	Continuous variables	OR (95% CI)	*P*
Infectious diseases	Fish types	Oily fish	0.986 5 (0.978 8, 0.994 3)	0.000 7
		Non-oily fish	1.010 1 (1.001 4, 1.018 8)	0.023 1
	Red meat types	Beef	1.034 7 (1.025 1, 1.044 4)	<0.000 1
		Mutton	1.061 4 (1.046 5, 1.076 5)	<0.000 1
		Pork	1.058 0 (1.043 7, 1.072 3)	<0.000 1
Respiratory infectious diseases	Fish types	Oily fish	0.989 9 (0.952 8, 1.027 6)	0.599 1
		Non-oily fish	1.035 1 (0.993 2, 1.077 8)	0.097 8
	Red meat types	Beef	1.037 0 (0.991 1, 1.083 7)	0.110 8
		Mutton	1.054 5 (0.985 1, 1.125 6)	0.118 8
		Pork	0.118 8 (1.006 8, 1.141 3)	0.026 9
Digestive infectious diseases	Fish types	Oily fish	0.979 4 (0.965 0, 0.993 8)	0.005 5
		Non-oily fish	0.999 8 (0.983 7, 1.016 1)	0.985 3
	Red meat types	Beef	1.024 7 (1.006 9, 1.042 7)	0.006 2
		Mutton	1.050 7 (1.023 4, 1.078 4)	0.000 2
		Pork	1.046 7 (1.020 4, 1.073 2)	0.000 4
Blood or sexually transmitted infectious diseases	Fish types	Oily fish	1.018 1 (0.960 7, 1.076 6)	0.536 2
		Non-oily fish	1.019 3 (0.957 0, 1.083 0)	0.544 2
	Red meat types	Beef	0.979 8 (0.913 5, 1.047 8)	0.560 4
		Mutton	1.025 0 (0.934 2, 1.118 3)	0.590 5
		Pork	1.097 9 (1.008 1, 1.189 2)	0.026 6

Multivariable logistic regression analysis was used and adjusted for age, sex, ethnicity, assessment center, activity, Townsend deprivation index, and education. Abbreviations: CI, confidence interval; OR, odds ratio.

Supplementary Table 7 **Associations between bread types/alcohol frequency and different types of infectious diseases in the UK Biobank**

Variables	Infectious diseases [OR (95% CI)*]	Respiratory infectious diseases [OR (95% CI)*]	Digestive infectious diseases [OR (95% CI)*]	Blood or sexually transmitted infectious diseases [OR (95% CI)*]
Bread types				
White	1.000 0 (Ref)	1.000 0 (Ref)	1.000 0 (Ref)	1.000 0 (Ref)
Brown	0.871 0 (0.847 4, 0.895 2)	0.819 0 (0.717 2, 0.932 9)	0.871 7 (0.828 2, 0.917 3)	1.193 0 (0.975 6, 1.453 5)
Whole meal or whole grain	0.775 2 (0.760 2, 0.790 5)	0.710 5 (0.647 2, 0.780 3)	0.788 4 (0.760 4, 0.817 6)	1.053 2 (0.902 9, 1.230 7)
Other type of bread	0.910 3 (0.872 5, 0.949 5)	0.871 7 (0.709 6, 1.060 5)	0.884 3 (0.817 1, 0.955 9)	1.444 8 (1.062 2, 1.927 9)
Alcohol frequency				
<Once a week	1.000 0 (Ref)	1.000 0 (Ref)	1.000 0 (Ref)	1.000 0 (Ref)
Once or twice a week	0.761 5 (0.745 1, 0.778 2)	0.689 0 (0.620 4, 0.764 5)	0.784 8 (0.754 2, 0.816 6)	0.673 7 (0.570 4, 0.793 7)
Three or four times a week	0.685 8 (0.669 9, 0.701 9)	0.645 3 (0.576 0, 0.721 9)	0.688 0 (0.658 5, 0.718 7)	0.513 0 (0.421 6, 0.621 0)
Daily or almost daily	0.756 3 (0.738 7, 0.774 4)	0.625 0 (0.555 2, 0.702 5)	0.740 7 (0.708 4, 0.774 2)	0.675 5 (0.560 6, 0.810 8)

Multivariable logistic regression analysis was used and adjusted for age, sex, ethnicity, assessment center, activity, Townsend deprivation index, and education. Abbreviations: CI, confidence interval; OR, odds ratio.

Supplementary Table 8 **Associations between main food groups and three infectious disease subgroups in the UK Biobank**

Reported consumption at recruitment	Participants (*n*)	Respiratory infectious diseases (*N*=2 663)			Digestive infectious diseases (*N*=18 678)			Blood or sexually transmitted (*N*=1 051)		
		Cases (*n*)	OR (95% CI)*	*P*	Cases (*n*)	OR (95% CI)*	*P*	Cases (*n*)	OR (95% CI)*	*P*
Processed meat										
Never	45 290	248	1.000 0 (Ref)		1671	1.000 0 (Ref)		135	1.000 0 (Ref)	
<1.0 time per week	147 928	762	0.961 1 (0.828 7, 1.118 9)	0.604 3	5514	1.003 5 (0.947 2, 1.063 7)	0.906 4	310	0.871 9 (0.707 3, 1.081 3)	0.205 0
1.0 time per week	141 849	788	1.011 9 (0.871 5, 1.179 3)	0.877 9	5364	1.020 4 (0.962 5, 1.082 4)	0.500 0	255	0.707 4 (0.568 2, 0.885 2)	0.002 2
⩾2.0 times per week	150 998	841	0.987 6 (0.849 3, 1.152 7)	0.872 9	6037	1.098 3 (1.035 8, 1.165 2)	0.001 8	341	0.727 5 (0.587 6, 0.906 1)	0.003 9
			P_{trend}=0.639 4			P_{trend}<0.000 1			P_{trend}=0.003 3	
Poultry										
Never	24 877	137	1.000 0 (Ref)		854	1.000 0 (Ref)		77	1.000 0 (Ref)	
<1.0 time per week	52 532	323	1.014 6 (0.824 7, 1.255 6)	0.892 7	2116	1.048 7 (0.964 3, 1.141 4)	0.269 0	152	0.989 9 (0.746 8, 1.323 6)	0.944 3
1.0 time per week	174 729	984	0.949 4 (0.788 9, 1.152 5)	0.590 9	6733	1.040 7 (0.965 6, 1.123 1)	0.300 9	335	0.732 7 (0.568 7, 0.956 6)	0.018 8
⩾2.0 times per week	234 074	1196	0.944 6 (0.787 1, 1.143 8)	0.549 5	8895	1.098 4 (1.020 4, 1.184 0)	0.013 4	481	0.750 9 (0.588 1, 0.973 0)	0.025 5
			P_{trend}=0.583 5			P_{trend}=0.000 1			P_{trend}=0.010 1	
Red meat										
<1 time per week	51 608	293	1.000 0 (Ref)		1889	1.000 0 (Ref)		161	1.000 0 (Ref)	
1.0–1.9 times per week	191 979	995	0.941 9 (0.821 1, 1.084 3)	0.398 5	7320	1.036 3 (0.981 8, 1.094 3)	0.197 8	366	0.737 4 (0.607 9, 0.898 9)	0.002 2
2.0–2.9 times per week	135 742	709	0.934 9 (0.809 5, 1.082 9)	0.364 3	5000	0.997 0 (0.942 1, 1.055 6)	0.918 8	247	0.737 3 (0.598 7, 0.910 8)	0.004 4
⩾3.0 times per week	108 520	666	1.015 9 (0.877 7, 1.179 2)	0.833 7	4469	1.096 3 (1.034 9, 1.161 8)	0.001 8	277	0.837 3 (0.682 4, 1.031 2)	0.091 5
			P_{trend}=0.029 7			P_{trend}<0.000 1			P_{trend}=0.386 2	
Red and processed meat										
<2.0 times per week	73 194	410	1.000 0 (Ref)		1889	1.000 0 (Ref)		190	1.000 0 (Ref)	
2.0–2.9 times per week	140 970	697	0.918 3 (0.808 3, 1.044 9)	0.193 2	7320	0.974 1 (0.927 5, 1.023 3)	0.295 0	266	0.912 9 (0.751 8, 1.111 0)	0.359 9
3.0–3.9 times per week	74 210	423	1.006 3 (0.871 8, 1.161 9)	0.931 1	5000	0.979 2 (0.925 8, 1.035 7)	0.462 8	150	0.926 9 (0.739 5, 1.159 8)	0.507 8
⩾4.0 times per week	199 475	1133	0.991 5 (0.878 4, 1.121 5)	0.891 1	4469	1.057 3 (1.008 8, 1.108 5)	0.020 4	445	0.849 9 (0.709 2, 1.022 6)	0.081 2
			P_{trend}=0.117 8			P_{trend}<0.000 1			P_{trend}=0.664 1	
Fish										
<1 time per week	40 368	276	1.000 0 (Ref)		1807	1.000 0 (Ref)		123	1.000 0 (Ref)	
1.0–1.9 times per week	194 838	998	0.786 5 (0.682 4, 0.909 9)	0.001 1	7219	0.853 1 (0.806 7, 0.902 7)	<0.000 1	402	0.913 1 (0.737 6, 1.140 0)	0.412 2

Supplementary Table 8 **Associations between main food groups and three infectious disease subgroups in the UK Biobank** (Continued)

Reported consumption at recruitment	Participants (n)	Respiratory infectious diseases (N=2 663)			Digestive infectious diseases (N=18 678)			Blood or sexually transmitted (N=1 051)		
		Cases (n)	OR (95% CI)*	P	Cases (n)	OR (95% CI)*	P	Cases (n)	OR (95% CI)*	P
2.0–2.9 times per week	114 736	594	0.763 5 (0.655 4, 0.892 2)	0.000 6	4236	0.824 9 (0.777 0, 0.876 2)	<0.000 1	208	0.910 4 (0.718 9, 1.159 5)	0.441 2
⩾3.0 times per week	137 907	795	0.835 2 (0.721 3, 0.970 7)	0.017 4	5416	0.861 3 (0.812 8, 0.913 3)	<0.000 1	318	1.055 4 (0.845 9, 1.326 8)	0.638 4
			P_{trend}=0.624 6			P_{trend}=0.058 4			P_{trend}=0.512 7	
Cheese										
<1.0 time per week	95 186	585	1.000 0 (Ref)		4178	1.000 0 (Ref)		237	1.000 0 (Ref)	
1.0 time per week	101 736	555	0.907 8 (0.804 1, 1.024 8)	0.117 8	4186	0.953 1 (0.910 6, 0.997 5)	0.038 7	203	0.948 6 (0.778 9, 1.154 5)	0.598 9
2.0–4.9 times per week	214 692	1108	0.919 7 (0.828 2, 1.022 5)	0.119 6	7647	0.877 1 (0.842 5, 0.913 3)	<0.000 1	412	0.990 7 (0.835 8, 1.177 5)	0.915 1
⩾5.0 times per week	62 660	293	0.884 3 (0.762 5, 1.023 5)	0.101 5	1946	0.823 8 (0.778 1, 0.872 0)	<0.000 1	146	1.166 8 (0.936 2, 1.450 3)	0.166 8
			P_{trend}=0.119 5			P_{trend}<0.000 1			P_{trend}=0.125 9	
Fruit										
<2.0 servings per day	162 372	959	1.000 0 (Ref)		6600	1.000 0 (Ref)		406	1.000 0 (Ref)	
2.0–2.9 servings per day	123 195	621	0.893 0 (0.803 6, 0.991 8)	0.035 1	4438	0.889 5 (0.854 4, 0.926 1)	<0.000 1	221	0.887 9 (0.748 9, 1.050 2)	0.168 1
3.0–3.9 servings per day	94 194	467	0.857 6 (0.763 2, 0.962 4)	0.009 4	3465	0.887 0 (0.848 9, 0.926 7)	<0.000 1	188	1.004 0 (0.836 3, 1.200 7)	0.965 7
⩾4.0 servings per day	108 088	616	0.948 2 (0.851 2, 1.055 5)	0.332 0	4175	0.912 5 (0.875 2, 0.951 3)	<0.000 1	236	1.050 3 (0.885 8, 1.242 4)	0.569 6
			P_{trend}=0.136 4			P_{trend}<0.000 1			P_{trend}=0.125 9	
Vegetables										
<2.0 servings per day	173 622	1000	1.000 0 (Ref)		6883	1.000 0 (Ref)		396	1.000 0 (Ref)	
2.0–2.9 servings per day	162 819	834	0.923 3 (0.838 7, 1.016 2)	0.102 9	5986	0.924 2 (0.890 8, 0.958 7)	<0.000 1	312	1.064 8 (0.912 1, 1.242 1)	0.425 2
3.0–3.9 servings per day	85 367	455	0.928 2 (0.826 0, 1.041 5)	0.207 7	3209	0.926 9 (0.886 6, 0.968 9)	0.000 8	166	1.053 9 (0.870 7, 1.270 0)	0.585 0
⩾4.0 servings per day	66 041	374	0.972 7 (0.857 7, 1.100 7)	0.663 9	2600	0.966 1 (0.920 7, 1.013 5)	0.159 5	177	1.247 1 (1.033 6, 1.498 5)	0.019 7
			P_{trend}=0.775 8			P_{trend}=0.049 2			P_{trend}=0.023 9	
Bread										
⩽5 slices a week	98 309	482	1.000 0 (Ref)		3647	1.000 0 (Ref)		203	1.000 0 (Ref)	
6–9 slices a week	91 692	523	1.090 9 (0.959 7, 1.240 4)	0.183 5	3528	1.001 5 (0.953 8, 1.051 6)	0.952 5	188	1.043 3 (0.847 8, 1.283 4)	0.688 6
10–14 slices a week	157 816	879	1.062 1 (0.946 2, 1.193 6)	0.309 3	6095	1.012 1 (0.968 9, 1.057 4)	0.589 2	318	1.025 1 (0.852 0, 1.236 6)	0.794 1

Supplementary Table 8 **Associations between main food groups and three infectious disease subgroups in the UK Biobank** (Continued)

Reported consumption at recruitment	Participants (*n*)	Respiratory infectious diseases (N=2 663)			Digestive infectious diseases (N=18 678)			Blood or sexually transmitted (N=1 051)		
		Cases (*n*)	OR (95% CI)*	*P*	Cases (*n*)	OR (95% CI)*	*P*	Cases (*n*)	OR (95% CI)*	*P*
≥15 slices a week	133 165	706	0.986 2 (0.870 6, 1.118)	0.827 5	5047	1.012 4 (0.966, 1.061)	0.607 4	307	1.003 0 (0.827 4, 1.218 9)	0.975 4
			P_{trend}=0.208 5			P_{trend}=0.208 5			P_{trend}=0.619 6	
Cereal										
≤1 bowl a week	103 382	600	1.000 0 (Ref)		4102	1.000 0 (Ref)		341	1.000 0 (Ref)	
2–4 bowls a week	97 722	562	0.990 9 (0.879 4, 1.116 3)	0.880 3	3921	1.019 3 (0.973 3, 1.067 5)	0.416 8	237	0.797 2 (0.670 9, 0.945 6)	0.009 6
5–6 bowls a week	99 724	466	0.829 4 (0.731 0, 0.940 5)	0.003 6	3506	0.918 1 (0.875 4, 0.962 7)	0.000 4	164	0.648 4 (0.533 7, 0.784 5)	<0.000 1
≥7 bowls a week	184 530	1011	0.919 5 (0.827 4, 1.022 8)	0.120 7	7037	0.935 5 (0.897 7, 0.974 9)	0.001 5	290	0.721 3 (0.611 3, 0.850 6)	0.000 1
			P_{trend}=0.021 9			P_{trend}<0.000 1			P_{trend}=0.000 3	
Tea										
<2.0 cups per day	127 477	682	1.000 0 (Ref)		4833	1.000 0 (Ref)		334	1.000 0 (Ref)	
2.0–3.9 cups per day	142 788	710	0.885 0 (0.794 0, 0.986 4)	0.027 3	5167	0.931 7 (0.894 0, 0.970 9)	0.000 8	304	0.872 7 (0.743 4, 1.024 1)	0.095 6
4.0–5.9 cups per day	123 853	673	0.943 4 (0.844 8, 1.053 4)	0.300 8	4688	0.948 1 (0.908 8, 0.989 1)	0.013 7	215	0.784 3 (0.656 3, 0.935 3)	0.007 1
≥6.0 cups per day	91 977	575	1.071 1 (0.954 3, 1.201 6)	0.242 5	3898	1.047 4 (1.001 7, 1.095 2)	0.041 8	188	0.858 5 (0.711 8, 1.032 2)	0.107 3
			P_{trend}=0.042 3			P_{trend}=0.004 0			P_{trend}=0.785 8	
Water										
<2 glasses per day	159 119	826	1.000 0 (Ref)		5937	1.000 0 (Ref)		286	1.000 0 (Ref)	
2 glasses per day	110 361	600	1.059 6 (0.950 1, 1.180 9)	0.296 9	4119	1.018 3 (0.976 7, 1.061 6)	0.394 5	206	1.043 1 (0.866 5, 1.253 3)	0.654 1
3–4 glasses per day	73 212	405	1.112 9 (0.983 4, 1.257 5)	0.088 0	2812	1.058 5 (1.009 4, 1.109 7)	0.018 6	148	1.111 6 (0.904 1, 1.360 9)	0.310 1
>4 glasses per day	140 888	797	1.154 7 (1.041 2, 1.280 5)	0.006 4	5599	1.129 5 (1.085 9, 1.174 8)	<0.000 1	391	1.308 2 (1.113 1, 1.539 1)	0.001 2
			P_{trend}=0.001 7			P_{trend}<0.000 1			P_{trend}<0.000 1	
Alcohol										
0 cup per day	152 326	1115	1.000 0 (Ref)		7440	1.000 0 (Ref)		496	1.000 0 (Ref)	
≤1 cup per day	140 746	597	0.655 5 (0.591 1, 0.726 4)	<0.000 1	4919	0.764 6 (0.735 5, 0.794 8)	<0.000 1	205	0.615 4 (0.517 1, 0.729 8)	<0.000 1
≤2 cups per day	106 735	488	0.653 4 (0.582 9, 0.731 4)	<0.000 1	3310	0.690 2 (0.660 5, 0.721 2)	<0.000 1	164	0.584 5 (0.484 1, 0.702 6)	<0.000 1
>2 cups per day	88 042	448	0.671 8 (0.594 9, 0.757 5)	<0.000 1	3009	0.752 6 (0.718 3, 0.788 4)	<0.000 1	186	0.658 6 (0.547 6, 0.789 1)	<0.000 1
			P_{trend}<0.000 1			P_{trend}<0.000 1			P_{trend}=0.039 6	

Multivariable logistic regression analysis was used and adjusted for age, sex, ethnicity, assessment center, activity, Townsend deprivation index, and education.
Abbreviations: CI, confidence interval; OR, odds ratio

Supplementary Table 9 **Associations between making dietary changes in the past five years and different types of infectious diseases in the UK Biobank**

Diseases	*N* (%)	OR (95% CI)	*P*
Infectious diseases			
Did not change	40 675 (54.40)	1.000 0 (Ref)	
Changed because of illness	14 834 (19.84)	2.109 0 (2.061 2, 2.157 7)	<0.000 1
Changed because of other reasons	19 263 (25.76)	1.058 6 (1.038 5, 1.079 1)	<0.000 1
Respiratory infectious diseases			
Did not change	1 394 (52.76)	1.000 0 (Ref)	
Changed because of illness	598 (22.63)	2.091 3 (1.888 4, 2.313)	<0.000 1
Changed because of other reasons	650 (24.60)	1.044 7 (0.948 8, 1.149 1)	0.370 9
Digestive infectious diseases			
Did not change	9 712 (52.26)	1.000 0 (Ref)	
Changed because of illness	3 973 (21.38)	2.120 8 (2.037 4, 2.207 1)	<0.000 1
Changed because of other reasons	4 899 (26.36)	1.103 2 (1.064 2, 1.143 6)	<0.000 1
Blood or sexually transmitted infectious diseases			
Did not change	544 (52.31)	1.000 0 (Ref)	
Changed because of illness	245 (23.56)	1.989 6 (1.692 6, 2.331 5)	<0.000 1
Changed because of other reasons	251 (24.13)	0.968 9 (0.830 4, 1.127 5)	0.685 6

Multivariable logistic regression analysis was used and adjusted for age, sex, ethnicity, assessment center, activity, Townsend deprivation index, and education.
Abbreviations: CI, confidence interval; OR, odds ratio.

Supplementary Table 10 **Associations between main food groups and infectious diseases in different ethnicities or races in the UK Biobank**

Reported consumption at recruitment	The White ethnicity or race (*N*=459 741)			The Asian or Asian British (*N*=4048)			The Black or Black British (*N*=7642)			Mixed ethnicity or race (*N*=16 418)		
	Participants (*n*)	Cases (*n*)	OR (95% CI)*	Participants (*n*)	Cases (*n*)	OR (95% CI)*	Participants (*n*)	Cases (*n*)	OR (95% CI)*	Participants (*n*)	Cases (*n*)	OR (95% CI)*
Processed meat												
Never	39 504	5 550	1.000 0 (Ref)	549	68	1.000 0 (Ref)	1 136	218	1.000 0 (Ref)	4 101	744	1.000 0 (Ref)
<1.0 time per week	138 713	19 878	0.978 4 (0.946 3, 1.011 7)	1 535	205	1.339 4 (0.973 5, 1.868 9)	2 873	456	0.920 7 (0.761 1, 1.116 9)	4 807	771	0.908 4 (0.805 9, 1.024 1)
1.0 time per week	135 904	20 738	1.016 9 (0.983 4, 1.051 7)	935	125	1.377 2 (0.974 0, 1.967 2)	1 742	332	1.126 3 (0.918 0, 1.384 5)	3 268	554	0.933 9 (0.817 1, 1.066 9)
≥2.0 times per week	144 937	24 096	1.101 0 (1.064 7, 1.138 8)	970	115	1.136 2 (0.792 8, 1.642 0)	1 744	321	1.102 2 (0.893 3, 1.362 3)	3 347	609	0.989 0 (0.865 2, 1.130 0)
			P_{trend}<0.000 1			P_{trend} = 0.829 6			P_{trend} = 0.074 0			P_{trend} = 0.889 2
Poultry												
Never	21 995	2 997	1.000 0 (Ref)	233	21	1.000 0 (Ref)	299	51	1.000 0 (Ref)	2 350	425	1.000 0 (Ref)
<1.0 time per week	49 781	8 408	1.051 0 (1.002 7, 1.101 9)	424	67	2.063 3 (1.196 3, 3.718 1)	603	109	1.141 1 (0.779 2, 1.690 0)	1 724	313	0.908 3 (0.760 2, 1.084 1)
1.0 time per week	167 287	26 153	1.011 1 (0.969 1, 1.055 3)	1 093	132	1.465 5 (0.884 7, 2.560 3)	1 841	344	1.115 6 (0.799 1, 1.585 5)	4 508	774	0.899 1 (0.779 3, 1.038 5)
≥2.0 times per week	219 915	32 678	1.040 1 (0.997 4, 1.085 0)	2 266	295	1.681 3 (1.039 4, 2.884 6)	4 834	842	1.077 6 (0.783 7, 1.511 5)	7 059	1 195	0.941 2 (0.822 8, 1.078 4)
			P_{trend}=0.006 7			P_{trend}=0.234 8			P_{trend}=0.870 2			P_{trend}=0.513 3
Red meat												
<1 time per week	45 047	6 430	1.000 0 (Ref)	569	81	1.000 0 (Ref)	959	171	1.000 0 (Ref)	5 033	977	1.000 0 (Ref)
1.0–1.9 times per week	182 598	26 979	0.993 5 (0.963 4, 1.024 7)	1 263	164	0.894 6 (0.653 7, 1.234 0)	2 764	450	0.885 8 (0.721 4, 1.092 1)	5 354	874	0.855 1 (0.762 1, 0.959 6)
2.0–2.9 times per week	131 103	19 787	0.996 7 (0.965 3, 1.029 1)	790	83	0.796 2 (0.556 9, 1.140 2)	1 524	276	1.026 1 (0.820 0, 1.287 5)	2 325	372	0.864 2 (0.746 9, 0.998 4)
≥3.0 times per week	100 993	17 237	1.101 8 (1.066 3, 1.138 5)	1 426	191	1.029 6 (0.756 2, 1.414 7)	2 395	458	1.095 5 (0.889 1, 1.355 2)	3 706	679	0.967 9 (0.853 4, 1.097 4)
			P_{trend}<0.000 1			P_{trend}=0.315 1			P_{trend}=0.212 4			P_{trend}=0.637 1
Red and processed meat												
<2.0 times per week	64 060	9 231	1.000 0 (Ref)	873	127	1.000 0 (Ref)	1 717	281	1.000 0 (Ref)	6 544	1 240	1.000 0 (Ref)
2.0–2.9 times per week	134 804	19 091	0.954 4 (0.928 0, 0.981 6)	898	107	0.843 2 (0.622 1, 1.141 1)	2 010	333	1.028 8 (0.855 3, 1.238 2)	3 258	509	0.859 4 (0.758 1, 0.973 2)
3.0–3.9 times per week	71 018	10 860	1.014 5 (0.983 0, 1.047 0)	551	59	0.822 8 (0.572 3, 1.171 0)	868	176	1.352 2 (1.080 0, 1.689 4)	1 773	294	0.884 1 (0.757 0, 1.029 7)
≥4.0 times per week	189 859	31 251	1.079 0 (1.050 4, 1.108 4)	1 726	226	0.987 6 (0.761 9, 1.285 9)	3 047	565	1.227 4 (1.034 5, 1.459 0)	4 843	859	0.957 4 (0.856 9, 1.069 3)
			P_{trend}<0.000 1			P_{trend}=0.613 6			P_{trend}=0.106 8			P_{trend}=0.995 9

Supplementary Table 10 **Associations between main food groups and infectious diseases in different ethnicities or races in the UK Biobank** (Continued)

Reported consumption at recruitment	The White ethnicity or race (*N*=459 741)			The Asian or Asian British (*N*=4048)			The Black or Black British (*N*=7642)			Mixed ethnicity or race (*N*=16 418)		
	Participants (*n*)	Cases (*n*)	OR (95% CI)*	Participants (*n*)	Cases (*n*)	OR (95% CI)*	Participants (*n*)	Cases (*n*)	OR (95% CI)*	Participants (*n*)	Cases (*n*)	OR (95% CI)*
Fish												
<1 time per week	35 382	6 106	1.000 0 (Ref)	374	41	1.000 0 (Ref)	556	91	1.000 0 (Ref)	4 056	828	1.000 0 (Ref)
1.0–1.9 times per week	184 710	27 593	0.854 5 (0.827 4, 0.882 6)	1 658	218	1.381 1 (0.929 8, 2.113 4)	2 701	472	1.082 9 (0.832 2, 1.424 5)	5 769	966	0.905 6 (0.801 8, 1.023 6)
2.0–2.9 times per week	109 622	16 235	0.814 2 (0.786 8, 0.842 7)	844	103	1.221 7 (0.794 7, 1.922 2)	1 407	251	1.037 2 (0.781 7, 1.388 2)	2 863	466	0.866 1 (0.749 9, 0.999 9)
≥3.0 times per week	130 027	20 499	0.864 1 (0.835 7, 0.893 6)	1 172	157	1.399 2 (0.931 2, 2.161 2)	2 978	541	0.955 5 (0.734 4, 1.256 5)	3 730	642	0.884 2 (0.773 3, 1.011 2)
			P_{trend}=0.276 1			P_{trend}=0.985 3			P_{trend}=0.723 5			P_{trend}=0.949 9
Cheese												
<1.0 time per week	85 208	14 388	1.000 0 (Ref)	1 648	224	1.000 0 (Ref)	3 364	607	1.000 0 (Ref)	4 966	915	1.000 0 (Ref)
1.0 time per week	95 646	15 324	0.960 6 (0.935 9, 0.985 9)	864	113	0.936 9 (0.718 0, 1.216 0)	1 625	277	0.979 7 (0.827 9, 1.156 8)	3 601	642	0.983 1 (0.869 5, 1.110 9)
2.0–4.9 times per week	207 192	30 339	0.921 4 (0.900 6, 0.942 6)	1 023	114	0.762 7 (0.587 1, 0.985 8)	1 690	270	0.935 0 (0.790 6, 1.103 5)	4 787	751	0.898 3 (0.800 4, 1.008 0)
≥5.0 times per week	60 617	7 965	0.882 7 (0.855 7, 0.910 5)	281	30	0.689 1 (0.428 2, 1.064 7)	312	53	0.978 9 (0.694 6, 1.351 9)	1 450	229	0.910 2 (0.767 4, 1.075 9)
			P_{trend}<0.000 1			P_{trend}=0.007 7			P_{trend}=0.582 5			P_{trend}=0.061 5
Fruit												
<2.0 servings per day	153 083	25 179	1.000 0 (Ref)	1 309	165	1.000 0 (Ref)	2 487	425	1.000 0 (Ref)	5 493	1 031	1.000 0 (Ref)
2.0–2.9 servings per day	117 272	17 042	0.891 5 (0.872 0, 0.911 5)	944	98	0.800 2 (0.597 3, 1.066 8)	1 586	258	0.881 3 (0.734 5, 1.055 4)	3 393	569	0.930 1 (0.819 2, 1.055 3)
3.0–3.9 servings per day	89 313	12 958	0.884 9 (0.863 7, 0.906 5)	724	93	1.032 0 (0.764 5, 1.386 1)	1 384	252	1.031 0 (0.856 2, 1.239 4)	2 773	444	0.862 5 (0.751 5, 0.988 5)
≥4.0 servings per day	100 073	15 254	0.910 8 (0.889 9, 0.932 2)	1 071	163	1.184 6 (0.912 3, 1.538 0)	2 185	420	0.990 7 (0.840 3, 1.167 7)	4 759	858	0.968 8 (0.862 7, 1.088 0)
			P_{trend}<0.000 1			P_{trend}=0.121 8			P_{trend}=0.520 8			P_{trend}=0.303 8
Vegetables												
<2.0 servings per day	163 637	25 914	1.000 0 (Ref)	1 236	150	1.000 0 (Ref)	3 078	540	1.000 0 (Ref)	5 671	1 098	1.000 0 (Ref)
2.0–2.9 servings per day	155 788	22 981	0.939 7 (0.920 9, 0.958 9)	1 070	152	1.189 6 (0.910 8, 1.554 6)	1 875	316	0.883 1 (0.747 9, 1.041 2)	4 086	659	0.830 5 (0.735 7, 0.937 1)
3.0–3.9 servings per day	80 670	12 226	0.952 3 (0.929 2, 0.975 9)	707	82	0.951 3 (0.692 8, 1.298 2)	1 271	228	0.979 5 (0.814 8, 1.174 5)	2 719	467	0.947 7 (0.829 0, 1.082 2)
≥4.0 servings per day	59 646	9 312	0.978 2 (0.952 0, 1.004 9)	1 035	135	1.075 0 (0.817 5, 1.413 1)	1 418	271	1.069 4 (0.898 1, 1.271 2)	3 942	678	0.912 5 (0.808 9, 1.029 0)
			P_{trend}=0.015 4			P_{trend}=0.714 2			P_{trend}=0.828 1			P_{trend}=0.011
Bread												
≤5 slices a week	89 205	12 506	1.000 0 (Ref)	1 365	177	1.000 0 (Ref)	2 893	508	1.000 0 (Ref)	4 846	828	1.000 0 (Ref)
6–9 slices a week	85 761	13 152	1.023 9 (0.996 1, 1.052 5)	839	114	0.946 7 (0.714 9, 1.248 1)	1 732	308	1.015 6 (0.859 0, 1.199 2)	3 360	551	0.895 8 (0.786 7, 1.019 3)

Supplementary Table 10 **Associations between main food groups and infectious diseases in different ethnicities or races in the UK Biobank** (Continued)

Reported consumption at recruitment	The White ethnicity or race (*N*=459 741)			The Asian or Asian British (*N*=4048)			The Black or Black British (*N*=7642)			Mixed ethnicity or race (*N*=16 418)		
	Participants (*n*)	Cases (*n*)	OR (95% CI)*	Participants (*n*)	Cases (*n*)	OR (95% CI)*	Participants (*n*)	Cases (*n*)	OR (95% CI)*	Participants (*n*)	Cases (*n*)	OR (95% CI)*
10–14 slices a week	150 615	22 979	0.998 4 (0.974 1, 1.023 4)	1 069	127	0.852 6 (0.652 5, 1.111)	1 759	315	0.992 0 (0.838 1, 1.172 7)	4 373	772	1.041 5 (0.925 1, 1.172 5)
≥15 slices a week	128 793	20 611	1.012 6 (0.986 6, 1.039 4)	684	89	0.986 2 (0.727 9, 1.328 9)	938	156	0.978 1 (0.786 2, 1.211 6)	2 750	492	1.022 2 (0.890 9, 1.171 9)
			P_{trend}=0.404			P_{trend}=0.895 2			P_{trend}=0.604 2			P_{trend}=0.039 5
Cereal												
≤1 bowl a week	94 034	15 565	1.000 0 (Ref)	1 649	230	1.000 0 (Ref)	2 486	455	1.000 0 (Ref)	5 213	937	1.000 0 (Ref)
2–4 bowls a week	89 587	14 254	0.964 8 (0.940 2, 0.990 1)	1 016	128	0.788 4 (0.608 4, 1.016 4)	2 655	478	0.925 2 (0.795 0, 1.076 8)	4 464	778	1.019 7 (0.909 5, 1.142 9)
5–6 bowls a week	95 002	12 992	0.843 1 (0.821 3, 0.865 6)	632	65	0.669 2 (0.486 1, 0.909 2)	1 279	211	0.892 2 (0.737 4, 1.076 6)	2 811	423	0.873 4 (0.762 1, 0.999 7)
≥7 bowls a week	180 028	27 375	0.870 6 (0.851 1, 0.890 5)	614	81	0.829 6 (0.616 3, 1.106 9)	1 024	183	0.885 8 (0.721 9, 1.083 1)	2 864	521	1.006 (0.883, 1.145 1)
			P_{trend}<0.000 1			P_{trend}=0.033			P_{trend}=0.226 6			P_{trend}=0.269 3
Tea												
<2.0 cups per day	119 470	18 488	1.000 0 (Ref)	1 106	143	1.000 0 (Ref)	2 794	472	1.000 0 (Ref)	4 107	675	1.000 0 (Ref)
2.0–3.9 cups per day	131 484	18 683	0.876 4 (0.856 6, 0.896 7)	1 555	190	0.841 1 (0.653 2, 1.084 6)	2 956	538	1.008 0 (0.871 6, 1.166 0)	6 793	1 198	0.975 6 (0.872 4, 1.091 7)
4.0–5.9 cups per day	118 433	17 718	0.901 3 (0.880 5, 0.922 5)	903	109	0.829 7 (0.621 1, 1.104 9)	1 294	230	0.981 3 (0.813, 1.181 2)	3 223	570	0.979 0 (0.856 9, 1.117 9)
≥6.0 cups per day	89 424	15 283	1.027 3 (1.002 4, 1.052 7)	467	73	1.266 8 (0.911 8, 1.747 6)	513	95	1.023 0 (0.782 1, 1.324 9)	1 573	286	1.053 7 (0.894 5, 1.238 6)
			P_{trend}<0.000 1			P_{trend}=0.621 3			P_{trend}=0.587			P_{trend}=0.760 8
Water												
<2 glasses per day	155 080	23 828	1.000 0 (Ref)	635	83	1.000 0 (Ref)	1 151	176	1.000 0 (Ref)	2 253	335	1.000 0 (Ref)
2 glasses per day	106 089	16 102	1.025 7 (1.002 8, 1.049 1)	657	66	0.746 9 (0.516 5, 1.076 1)	1 252	197	0.968 6 (0.765 6, 1.226 3)	2 363	386	1.101 2 (0.929 2, 1.305 5)
3–4 glasses per day	68 929	10 385	1.048 5 (1.021 5, 1.076 2)	715	85	0.891 7 (0.630 6, 1.261 3)	1 210	201	1.042 2 (0.823 8, 1.319 7)	2 358	405	1.176 3 (0.993 7, 1.393)
>4 glasses per day	126 519	19 485	1.143 1 (1.118 5, 1.168 2)	1 985	279	1.062 5 (0.802 1, 1.421 1)	3 810	730	1.189 5 (0.981 2, 1.449 1)	8 574	1 572	1.256 3 (1.096 5, 1.443 2)
			P_{trend}<0.000 1			P_{trend}=0.122 5			P_{trend}=0.000 8			P_{trend}=0.000 1
Alcohol_intake												
0 cups per day	133 963	25 398	1.000 0 (Ref)	2 621	352	1.000 0 (Ref)	4 948	924	1.000 0 (Ref)	10 794	2 109	1.000 0 (Ref)
≤1 cup per day	135 583	18 144	0.727 1 (0.711 4, 0.743 2)	801	87	0.804 3 (0.612 4, 1.046 3)	1 529	239	0.842 5 (0.711 7, 0.993 9)	2 833	415	0.726 0 (0.639 4, 0.822 7)
≤2 cups per day	104 090	13 711	0.699 5 (0.683, 0.716 4)	365	41	0.804 8 (0.551 7, 1.146 3)	716	120	0.915 5 (0.729 5, 1.140 2)	1 564	194	0.591 0 (0.497 4, 0.698 5)
>2 cups per day	86 105	13 180	0.788 9 (0.769 4, 0.808 8)	261	39	1.055 1 (0.708 2, 1.535 7)	449	72	0.804 3 (0.597 5, 1.066 2)	1 227	184	0.735 7 (0.615 2, 0.875 3)
			P_{trend}<0.000 1			P_{trend}=0.259			P_{trend}=0.218 8			P_{trend}=0.001 5

Multivariable logistic regression analysis was used and adjusted for age, sex, ethnicity, assessment center, activity, Townsend deprivation index, and education.
Abbreviations: CI, confidence interval; OR: odds ratio.

***Supplementary Table 11* Association between four diet groups and infectious diseases**

Outcome variables	Four diet groups	Participants (*n*)	Cases (*n*)	OR (95% CI)	*P*
Infectious diseases	Regular meat-eaters	282 066	44 587	1.000 0 (Ref)	
	Low meat-eaters	184 201	27 802	0.940 4 (0.924 3, 0.956 7)	<0.000 1
	Fish-eaters	11 012	1269	0.839 1 (0.788 7, 0.891 9)	<0.000 1
	Vegetarians	8933	1146	0.915 4 (0.856 1, 0.977 8)	0.009 1
Respiratory infectious diseases	Regular meat-eaters	282 066	1554	1.000 0 (Ref)	
	Low meat-eaters	184 201	990	0.973 7 (0.895 6, 1.058 2)	0.531 5
	Fish-eaters	11 012	45	0.815 6 (0.587 2, 1.099 3)	0.201 4
	Vegetarians	8933	51	1.152 0 (0.845 8, 1.529 5)	0.348 0
Digestive infectious diseases	Regular meat-eaters	282 066	10 865	1.000 0 (Ref)	
	Low meat-eaters	184 201	7134	0.965 1 (0.934 8, 0.996 4)	0.029 2
	Fish-eaters	11 012	325	0.852 5 (0.758 8, 0.954 1)	0.006 3
	Vegetarians	8933	274	0.861 7 (0.757 4, 0.976 0)	0.021 4
Blood or sexually transmitted infectious diseases	Regular meat-eaters	282 066	610	1.000 0 (Ref)	
	Low meat-eaters	184 201	376	1.015 5 (0.886 7, 1.161 3)	0.823 5
	Fish-eaters	11 012	33	1.389 0 (0.944 0, 1.967 0)	0.078 4
	Vegetarians	8933	26	1.197 8 (0.777 9, 1.757 9)	0.383 6

Multivariable logistic regression analysis was used and adjusted for age, sex, ethnicity, assessment center, activity, Townsend deprivation index, and education. Abbreviations: CI, confidence interval; OR, odds ratio.

***Supplementary Table 12* Mediation effects of diet groups on infectious diseases by potential variables in UKB**

Outcome	Mediator	Exposure	Total effect (OR with 95% CI)*	Direct effect (OR with 95% CI)*	Mediation proportion (%) (95% CI)*	*P*
Infectious diseases	TG	Low meat-eaters *vs.* ref.	0.950 2 (0.933 5, 0.967 1)	0.993 6 (0.991 4, 0.995 8)	14.19 (10.43, 20.61)	<0.000 1
		Fish-eaters *vs.* ref.	0.861 1 (0.808 1, 0.916 8)	0.982 4 (0.975 4, 0.989 5)	7.92 (5.41, 12.68)	<0.000 1
	Glucose	Low meat-eaters *vs.* ref.	0.949 8 (0.932 4, 0.967 6)	0.993 6 (0.991 2, 0.995 8)	7.27 (5.21, 10.99)	<0.000 1
		Fish-eaters *vs.* ref.	0.846 2 (0.791 3, 0.904 0)	0.980 4 (0.973 7, 0.987 9)	5.37 (3.63, 8.70)	<0.000 1
	HbA1c	Low meat-eaters *vs.* ref.	0.948 9 (0.932 2, 0.965 9)	0.993 5 (0.991 3, 0.995 7)	17.26 (13.42, 24.18)	<0.000 1
		Fish-eaters *vs.* ref.	0.871 0 (0.817 1, 0.927 5)	0.983 6 (0.977 2, 0.990 7)	12.75 (9.21, 21.41)	<0.000 1
	WBC	Low meat-eaters *vs.* ref.	0.954 5 (0.937 9, 0.971 4)	0.994 2 (0.992 2, 0.996 4)	24.14 (18.53, 34.1)	<0.000 1
		Fish-eaters *vs.* ref.	0.876 2 (0.822 5, 0.932 6)	0.984 3 (0.977 5, 0.991 3)	20.63 (14.37, 32.13)	<0.000 1
	BMI	Low meat-eaters *vs.* ref.	0.972 5 (0.955 7, 0.989 6)	0.996 5 (0.994 3, 0.998 7)	53.59 (41.18, 75.45)	<0.000 1
		Fish-eaters *vs.* ref.	0.911 6 (0.856 1, 0.969 8)	0.989 3 (0.982 8, 0.996 7)	44.87 (33.01, 73.12)	<0.000 1
	WHR	Low meat-eaters *vs.* ref.	0.970 3 (0.953 6, 0.987 3)	0.996 3 (0.994 3, 0.998 4)	50.41 (39.72, 70.67)	<0.000 1
		Fish-eaters *vs.* ref.	0.900 9 (0.846 3, 0.958 3)	0.987 7 (0.980 1, 0.995 1)	36.57 (25.73, 59.84)	<0.000 1
	Body fat percentage	Low meat-eaters *vs.* ref.	0.968 1 (0.951 2, 0.985 2)	0.996 0 (0.994 0, 0.998 1)	49.26 (38.37, 67.43)	<0.000 1
		Fish-eaters *vs.* ref.	0.917 5 (0.861 3, 0.976 5)	0.989 9 (0.982 4, 0.997 1)	47.39 (33.09, 77.04)	<0.000 1
	Whole body fat mass	Low meat-eaters *vs.* ref.	0.971 7 (0.954 8, 0.989 0)	0.996 5 (0.994 3, 0.998 7)	53.45 (41.84, 76.09)	<0.000 1
		Fish-eaters *vs.* ref.	0.919 1 (0.862 8, 0.978 3)	0.99 (0.983 2, 0.997 5)	46.87 (33.3, 77.84)	<0.000 1
	Arms fat mass	Low meat-eaters *vs.* ref.	0.964 5 (0.948 0, 0.981 4)	0.995 5 (0.993 3, 0.997 6)	39.69 (30.44, 55.78)	<0.000 1
		Fish-eaters *vs.* ref.	0.893 2 (0.839 2, 0.949 9)	0.986 6 (0.979 5, 0.993 8)	31.54 (22.82, 51.12)	<0.000 1
	Leg fat mass	Low meat-eaters *vs.* ref.	0.961 4 (0.944 9, 0.978 2)	0.995 2 (0.993 1, 0.997 4)	34.99 (26.95, 51.17)	<0.000 1
		Fish-eaters *vs.* ref.	0.887 9 (0.834 2, 0.944 2)	0.986 1 (0.979 3, 0.993 4)	28.44 (20.15, 47.18)	<0.000 1
	Trunk fat mass	Low meat-eaters *vs.* ref.	0.969 1 (0.952 2, 0.986 3)	0.996 1 (0.993 9, 0.998 2)	49.49 (38.15, 68.13)	<0.000 1
		Fish-eaters *vs.* ref.	0.910 7 (0.855 0, 0.969 2)	0.989 3 (0.982 3, 0.995 9)	42.58 (30.17, 66.34)	<0.000 1

*Adjusted for age, sex, ethnicity, assessment center, activity, Townsend deprivation index, and education.
Abbreviations: CI, confidence interval; OR, odds ratio; TG, total cholesterol; HbA1c, glycosylated hemoglobin; WBC, white blood cells; BMI, body mass index; WHR, waist-to-hip ratio.
